# A Small Sugar Molecule with Huge Potential in Targeted Cancer Therapy

**DOI:** 10.3390/pharmaceutics15030913

**Published:** 2023-03-11

**Authors:** Gabriela Pastuch-Gawołek, Julia Szreder, Monika Domińska, Mateusz Pielok, Piotr Cichy, Mirosława Grymel

**Affiliations:** 1 Department of Organic Chemistry, Bioorganic Chemistry and Biotechnology, Silesian University of Technology, B. Krzywoustego 4, 44-100 Gliwice, Poland; 2 Biotechnology Centre, Silesian University of Technology, B. Krzywoustego 8, 44-100 Gliwice, Poland

**Keywords:** targeted cancer therapy, prodrug, glycoconjugates, drug carriers, glycopolymers

## Abstract

The number of cancer-related diseases is still growing. Despite the availability of a large number of anticancer drugs, the ideal drug is still being sought that would be effective, selective, and overcome the effect of multidrug resistance. Therefore, researchers are still looking for ways to improve the properties of already-used chemotherapeutics. One of the possibilities is the development of targeted therapies. The use of prodrugs that release the bioactive substance only under the influence of factors characteristic of the tumor microenvironment makes it possible to deliver the drug precisely to the cancer cells. Obtaining such compounds is possible by coupling a therapeutic agent with a ligand targeting receptors, to which the attached ligand shows affinity and is overexpressed in cancer cells. Another way is to encapsulate the drug in a carrier that is stable in physiological conditions and sensitive to conditions of the tumor microenvironment. Such a carrier can be directed by attaching to it a ligand recognized by receptors typical of tumor cells. Sugars seem to be ideal ligands for obtaining prodrugs targeted at receptors overexpressed in cancer cells. They can also be ligands modifying polymers’ drug carriers. Furthermore, polysaccharides can act as selective nanocarriers for numerous chemotherapeutics. The proof of this thesis is the huge number of papers devoted to their use for modification or targeted transport of anticancer compounds. In this work, selected examples of broad-defined sugars application for improving the properties of both already-used drugs and substances exhibiting anticancer activity are presented.

## 1. Introduction

Due to the fact that in the last 60 years, sanitary conditions in the world have improved significantly, and many effective vaccines and antibiotics have been introduced to the market, the mortality rate caused by infectious diseases has significantly decreased. The decrease in the mortality rate of infectious diseases caused cancer diseases, together with cardiovascular diseases, to become the main causes of death worldwide. In up to 127 out of 189 countries that were taken into account when preparing the statistics, these diseases were the main cause of death of people under 70, and cancer is the first or second leading cause of death in as many as 112 of them [1,2]. This applies primarily to countries with a high Human Development Index (HDI) and is also largely related to the aging of the population. According to the latest report by the International Agency for Research on Cancer (IARC), in 2020, there were 19.3 million new cases of cancer worldwide and almost 10 million deaths [3]. In the case of women, breast cancer was the most frequently diagnosed cancer and one of the main causes of death. In turn, in men, the dominant cancers were lung and prostate cancers. It is estimated that by 2040 the number of new cancer cases will increase to 28.4 million [2].

Despite the continued development of science, effective treatment of some types of cancer is still difficult and sometimes even impossible. There are many therapeutic strategies used to treat cancer, including surgery, radiotherapy, and chemotherapy, but in many cases, they are not effective enough [4,5]. The low selectivity of most currently used chemotherapeutic agents results in drug accumulation in healthy tissue and systemic toxicity, which leads to serious side effects of applied therapies [6]. Another obstacle is the increasing resistance of cancer cells to drugs used, which significantly limits the successful results of anticancer therapy [7]. Therefore, researchers are still looking for ways to improve the properties of chemotherapeutic agents by reducing their systemic toxicity and increasing the selectivity profile.

**Figure 1:**
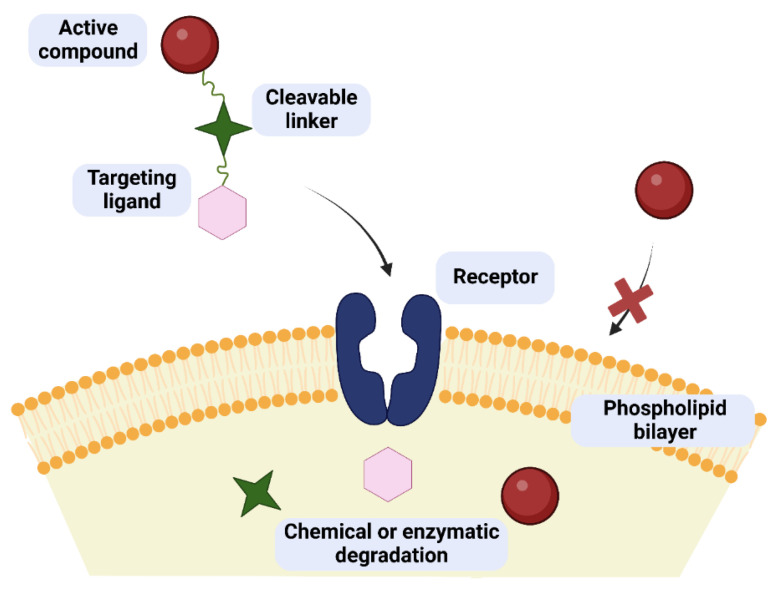
Concept of drug/glycoconjugate targeting in cancer therapy. The targeted drug can cross the cell membrane as opposed to the pure active compound. Inside the cell, the prodrug breaks down into its component parts.

One of the possibilities to improve the effectiveness and safety of chemotherapeutic agents is the development of targeted therapies that will allow the drug to selectively interact with cancer cells. The use of targeted drugs makes it possible to deliver the biologically active substance precisely to the pathologically affected site, preventing the uptake of such a drug by healthy cells and, consequently, reducing systemic toxicity. In such a case, it is possible to reduce the therapeutic dose of the drug to achieve its sufficient intracellular concentration compared to the traditional applied drugs. Designed molecules typically target specific enzymes, receptor proteins, and signaling pathways [8,9]. Obtaining such compounds is possible by covalent coupling of a therapeutic agent with an appropriate ligand (e.g., sugar, peptide, vitamin, protein, or antibody) acting as a selective transporter. The compound created in this way should act selectively by targeting receptors on the membrane surface of diseased cells, to which the attached targeting ligand shows affinity and to which expression in the case of diseased cells is much higher than normal cells [10]. The success of such an approach depends to a large extent on the binding capacity of the ligand to the potential receptor, as well as the stability of the prodrug in the systemic circulation and its no-degradation before reaching target cells [11,12]. The structure of a targeted drug usually consists of several elements, such as a targeting ligand, a linker, and a specific active molecule (Figure 1). Each of these fragments should be meticulously designed because the effectiveness of the entire drug will depend on each of them. The targeting ligand plays a key role in the selective delivery of the drug to target cells that overexpress specific receptors. Its effectiveness is modulated by its specificity for tumor cells compared to normal cells. The linker between the ligand and the therapeutic payload should be designed to ensure the stability of the molecule in the systemic circulation and be easily cleaved upon reaching the target cells, thus releasing the attached drug responsible for exerting a specific pharmacological effect [10,11,12].

**Scheme 1:**
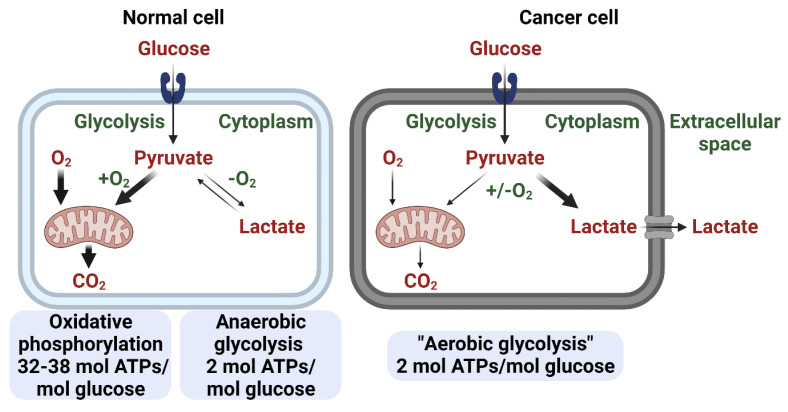
Glucose metabolism in healthy and cancer-transformed cells. Normal cells convert sugar by glycolysis, followed by the Krebs cycle (high ATP yield). When there is insufficient oxygen, the stored pyruvic acid in the cell is converted to lactic acid. In cancer cells, even in the presence of oxygen, metabolism proceeds in the direction of lactic acid production, resulting in low ATP gain (Warburg effect, ‘aerobic glycolysis’). Excretion of lactic acid out of the cell is one of the causes of the reduced pH of the tumor microenvironment [31,32].

An important factor to consider when designing prodrugs for use in targeted anticancer therapies is the environment in which cancer cells grow. The tumor microenvironment, which supports the survival and development of cancer cells, is characterized by low oxygen concentration [13], changed pH [14], increased levels of reactive oxygen species (ROS) [15] and glutathione [16], increased demand for certain micronutrients [17], and overexpression of specific enzymes [18]. These properties play an essential role in cancer cells progression and metastasis and are a valuable clue in the development of new cancer targeting therapies [19]. A very important difference between cancer cells and normal cells is their dissimilar glucose metabolism. It has been observed that cancer cells show increased uptake of glucose compared to healthy cells and also metabolize it in a specific way in order to obtain the energy needed to enhance proliferation. This observation is known as the Warburg effect and is the result of mitochondrial metabolic changes [20]. The consequence of the aforementioned hypoxia is that cancer cells, unlike healthy cells, for which the main source of energy is mitochondrial oxidative phosphorylation, produce energy through so-called ‘aerobic glycolysis’. This situation occurs even in the presence of oxygen, and the consequence is the production of large amounts of lactate (Scheme 1) [21]. It may seem that in terms of ATP production, cancer cells have developed a less efficient metabolism; however, the rate of glucose metabolism in cancer cells is enormous, which compensates for the small energy gain (amount of ATP produced) relative to oxidative phosphorylation [21,22]. The increased glycolysis process is associated with a greater demand for glucose in cells, which is accompanied by the overexpression of sugar transporters in cancer cells. Sugars can be transported into cells through two families of transporters: sodium-coupled glucose transporters (SGLTs) and glucose facilitative transporters (GLUTs). GLUTs are special transmembrane proteins that mediate the energy-independent active transport of sugar molecules into the cell. They are divided into class I, II, and III, and among them, GLUT1, encoded by the SLC2A1 gene, is the best characterized and most frequently overexpressed transporter in many human cancer cells [23]. It can bind to glucose, galactose, mannose, glucosamine, or ascorbic acid and then transport these molecules across cell membranes [24]. Many studies have shown that high levels of GLUT1 expression are strongly correlated with a poor prognosis in many cancers [25,26,27,28]. This unique property is the basis for imaging techniques, such as positron emission tomography (PET), which tracks the radioactively labeled glucose analogue, [^18^F]-2-fluoro-2-deoxy-D-glucose (^18^F-FDG). It is a widely used diagnostic tool to visualize tumor tissues and their metastases due to the tendency of tumor cells to take up glucose at a faster rate than most normal tissues [29]. The high uptake of glucose or other sugars by cancer cells makes sugar conjugates with biologically active molecules seem to be good candidates for targeted drugs. In this way, glycoconjugates can be preferentially taken up by cancer cells and only minimally reach healthy cells [30].

The second solution used to improve the properties of anticancer drugs is the use of various types of carriers that enable the controlled release of the drug under the influence of factors characteristic of the tumor microenvironment. Their use should allow the protection of the active substance against premature lysosomal degradation and reaction in the biological environment. Additionally, their use should enable the control of the pharmacokinetic profile and distribution of the chemotherapeutic agent, extend the time of its circulation in the bloodstream, which leads to an increase in the efficiency of access to cancer cells, and enable the administration of hydrophobic drugs by intravenous route. Such carriers include liposomes, polymer micelles, nanogels, or carbon nanotubes [33,34]. Carriers of this type can be, for example, glycopolymers which, having a sugar moiety attached, can be easily delivered to the tumor because of the Warburg effect and overexpression of GLUT transporters or affinity for cancer cell lectins. It should be taken into account that the mechanism of GLUT-mediated uptake in the case of glycosylated small molecules and much larger macromolecular conjugates or glycosylated nanocarriers will be significantly different. In the case of small glycoconjugates, they bind to the transporter, changing its geometry, and are transported across the membrane and released into the cell. In the case of glycosylated nanocarriers, a mechanism is postulated in which a sugar ligand bounds to a transporter, which initiates conformational changes leading to the initiation of endocytosis proceeding through various pathways [23]. Due to factors specific to the tumor microenvironment, controlled release of the drug from such carriers is possible [35]. An interesting solution is also the use of polysaccharides, such as alginate [36], hyaluronic acid [37], heparin (sulfate) [38,39], carrageenan [40], dextran [41], chitin, or chitosan [42], to create drug-delivery nanoparticles. These compounds are characterized by biocompatibility and often also turn out to be bioactive [43].

Sugars, which are a structural element during both the synthesis of the aforementioned glycoconjugates and the preparation of polysaccharide or glycopolymer carriers of anticancer drugs, are widely distributed in nature and also constitute one of the main classes of natural compounds found in living organisms, where they perform important functions in many physiological and pathological processes [44]. For this reason, in this work, it was decided to present a number of examples of their use to improve the properties of both already used therapeutic agents as well as compounds characterized by cytotoxicity which allows for inhibiting the proliferation of cancer cells.

## 2. Glycoconjugates

The development of glycobiology, i.e., the science that studies the structure and function of sugars and their connections, has allowed the discovery of the significant therapeutic potential of these compounds. Glycoconjugation, which is understood to be the connection of sugar derivatives with another compound by creating a covalent bond, is widely used in the design of new derivatives that support the fight against various diseases, including cancer [45]. The glycoconjugation strategy aims to improve the bioavailability, selectivity, and solubility of potential drugs in biological systems. Sugar-based prodrugs are of great interest in cancer treatment due to the possibility of their targeted delivery to cancer cells characterized by increased demand for glucose and the associated overexpression of protein receptors responsible for the transport of sugars into the cell [30]. The best proof of the attention paid to this subject is provided by the number of reports on the glycoconjugation of various active molecules that can be found in the scientific literature. After entering the entry ‘glycoconjugates’ in the *Scopus database*, nearly eleven thousand literature items appear, of which one-third have appeared in the last ten years. Numerous glycoconjugates of biologically active compounds, both drugs already approved for use in anticancer therapy, as well as substances just being tested for their potential use in cancer treatment, turned out to be promising molecules with better solubility, reduced systemic toxicity, and increased activity in relation to parent compounds, both in routine in vitro cytotoxicity studies and in in vivo laboratory studies in animal models.

When designing the structure of glycoconjugates, isomers that can be formed (positions in a sugar used to form a connection with a biologically active compound) must be taken into account. This is important from the point of view of creating stable interactions between the glycoconjugate and GLUT1. Scientists can find many papers related to this issue and point to the importance of substituting individual positions in the sugar on the affinity of the resulting conjugate for GLUT transporters. The conclusions presented in them are not always consistent; however, the majority of papers describe that glycoconjugates formed using the C1 position in sugar. It can be assumed that this is because it is easiest to functionalize the sugar in the anomeric position. It is worth noting that, in most cases where the obtained compounds showed biological activity, the substituent at the C1 position of the sugar was in the equatorial position. Based on the literature reports, it can be concluded that hydroxyl groups at C2, C4, and C6 are not involved in hydrogen bonding with GLUT1. Therefore, the attachment of biologically active compounds at these sugar positions should not reduce the affinity of the obtained glycoconjugates for GLUT transporters. For glycoconjugation, not only D-glucose but also other sugars can be used, e.g., D-galactose, D-mannose, L-arabinose, D-ribose, or D-fucose, and to a lesser extent, L-rhamnose and D-xylose. The type of sugar may be important when a certain type of cancer has high levels of specific hydrolytic enzymes, e.g., galactosidases, capable of releasing the biologically active compound from the prodrug [23].

**Scheme 2:**
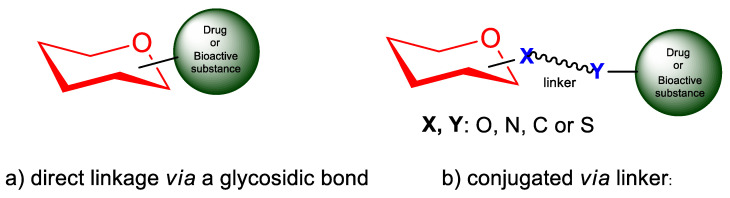
General structure of drug glycoconjugates and bioactive compounds conjugated sugar.

The library of glycoconjugates presented in our work can be divided according to the criterion of the bioactive agent used: (i) hybrids of sugars with known and used anticancer drugs and (ii) combinations of sugars with bioactive substances, often natural. The second criterion is the way in which the sugar moiety combines with the active unit. A variety of strategies have been described in the literature, including (a) direct linking via a glycosidic bond and (b) conjugating via a linkers (e.g., esters, amides, ureas, or succinic acid), as shown in Scheme 2.

### 2.1. Anticancer Drug Glycoconjugates

Despite significant advances in the diagnosis and treatment of cancer diseases, drug resistance still remains one of the main causes hampering the effectiveness of therapy. The emergence of resistance to therapy can occur at an early or later stage of treatment, thus limiting its success. It is important to understand the importance of altered glucose metabolism in driving cancer progression, response to treatment, and its role in resistance to commonly used drugs, such as doxorubicin, cisplatin, paclitaxel, and methotrexate, among others.

The first substance of natural origin used as an anticancer agent was podophyllotoxin (PDX). It is a potent anticancer agent but too toxic to be useful in the treatment of human neoplasms. Among many natural and synthetic derivatives of podophyllotoxin, two 4-demethylepipodophyllotoxin attached through *O*-glycosidic bond β-D-glucopyranoside cyclic acetals, known as etoposide (VP16-213) and teniposide (VM26), deserve attention. Teniposide is used in the treatment of childhood lymphoblastic leukemia, Hodgkin’s disease, non-Hodgkin lymphomas, and neuroblastoma. On the other hand, etoposide has activity against of testicular cancer, lymphomas, lung cancer, monocytic leukemia, non-Hodgkin lymphomas, and hepatocellular carcinoma [46].

The 1960s was a significant decade for the discovery of new anticancer drugs. During this period, Wani and Wallh isolated paclitaxel and the alkaloid, camptothecin. Camptothecin, obtained from extracts of *Camptotheca acuminata Decne* (in 1873), showed good activity against L1210 leukemia, but, unfortunately, there are major limitations to its use as an anticancer agent, including toxicity, nonselectivity, and inactivation by human serum albumin (HSA). The search for improved analogs led to the discovery of topotecan (*Hycamtin*, *N*,*N*-dimethylaminomethyl substituent at the C-9 position of the parent structure) and irinotecan (*Camptosar*, prodrug of the 7-ethyl-10-hydroxycamptothecin analog), which were approved for clinical use [46].

One of the most effective and widely used anticancer drugs is paclitaxel (PTX, *Taxol^®^*), isolated from the bark of *Taxus brevifolia* (*Pacific Yew*) and now also produced synthetically [47]. The first of several FDA approvals of various uses for *Taxol^®^* was announced in 1992 [48]. It is approved for the treatment of breast and ovarian cancers, AIDS-related Kaposi sarcoma, and a number of other cancers [47]. Although the initial response to paclitaxel is impressive, most breast cancer (BC) patients develop resistance, ultimately leading to relapse, metastasis, and death [49]. Unfortunately, paclitaxel has low oral bioavailability and very low selectivity [50,51]. The development of resistance of tumor cells and severe side effects in patients require further improvement of this drug.

An important class of chemotherapy drugs comprises anthracyclines, containing planar aromatic quinone rings connected to a sugar moiety. Doxorubicin (DOX), the active compound in the trade drug named adriamycin (ADM) and daunorubicin, which can be isolated from the bacterial strain *Streptomyces peucetius*, and epirubicin, idarubicin (semi-synthetic analog) belong to the most well-known antibiotics of the anthracycline family and are among the most prescribed drugs for the treatment of hematologic malignancies and adult solid tumors [52]. DOX, recognized by the *World Health Organization* (*WHO*), shows efficacy against carcinomas of the breast, ovary, bladder, stomach, and thyroid, as well as small cell lung cancer, soft-tissue and osteogenous sarcoma, and numerous solid paediatric tumors. DOX demonstrates activity against hematopoietic malignancies, such as leukemias, lymphomas (Hodgkin and non-Hodgkin’s), and multiple myeloma. It is also the standard against which the cytotoxicity of new potential therapeutic agents is evaluated. Despite the therapy of DOX increasing survival in patients, it can also lead to many side effects, such as nephrotoxicity, hepatotoxicity, bone marrow suppression, irreversible cardiotoxicity, drug-induced leukemia, thrombocytopenia, anemia, liver toxicity, myelosuppression, vomiting, alopecia, and ulcerative stomatitis [53].

Since 1978, Pt-based antitumor drugs (cisplatin, carboplatin, and oxaliplatin) have been the first choice of chemotherapy drugs for malignant tumors, such as testicular, colorectal, non-small cell lung, ovarian, breast, head and neck, and nasopharyngeal cancer. However, their application is limited by severe side effects, such as renal toxicity, ototoxicity, neurotoxicity, and alopecia, as well as the problems of drug and cross-resistance and intrinsic or acquired resistance [54]. Oxaliplatin (*Eloxatins^®^*) is widely used to treat colorectal cancer in combination with 5-fluorouracil (5-FU) and leucovorin (LV). Unfortunately, oxaliplatin also causes many side effects, such as gastrointestinal toxicity, bone marrow suppression, and neuro- or nephrotoxicity. The low solubility in water and the slow excretion of oxaliplatin are the causes of the accumulation of metals [55,56].

Modern drug design often ensures selective and effective drug delivery to cancer cells with less toxicity to healthy cells. The unique glucose metabolism of cancer cells is the driving force behind the development of anticancer drug glycoconjugates (ADGs) designed for selective uptake by cancer cells. As a result of the high solubility in water, low toxicity, and high biocompatibility, the sugar moiety is an attractive system to facilitate drug delivery. Glycoconjugation is a very good method of imparting increased aqueous solubility to hydrophobic scaffolds, including several drugs such as aspirin [57], warfarin [58], and oxaliplatin [59].

So far, many semi-synthetic derivatives of anticancer drugs have been synthesized, and their effect on cytotoxicity has been investigated in terms of structure–activity relationships (SARs). During 2000 to 2021, fifty-four carbohydrate-based agents that contain sugar moieties as the major structural units have been approved as drugs or diagnostic agents, including three (*Ambrucin*, Japan, 2002; *Mifamurtide*, 2009, Europe, and *Midostaurin*, USA, 2017) with anticancer activity [60]. Glycosylation of therapeutic agents many times has been found to improve their pharmacokinetic parameters, reduce adverse effects, and expand half-life compared to the parent (not glycosylated agent). In this chapter, we present the selected glycosylated therapeutic agents and the effect of attached sugar derivatives on the anticancer activity of those glycoconjugates.

The first glycoconjugate targeting GLUT1 transporters described in 1995 was glufosfamide, wherein β-D-glucose was connected to an alkylating agent, ifosfamide [61,62]. The task of the prodrug designed in this way was to increase the selectivity of ifosfamide (DNA alkylating drug) and reduction its toxicity [62]. In 1997, the first human clinical trials of glufosfamide were conducted in Europe [63]. A little later, research was continued in Japan and the USA, with promising results [64].

**Figure 2:**
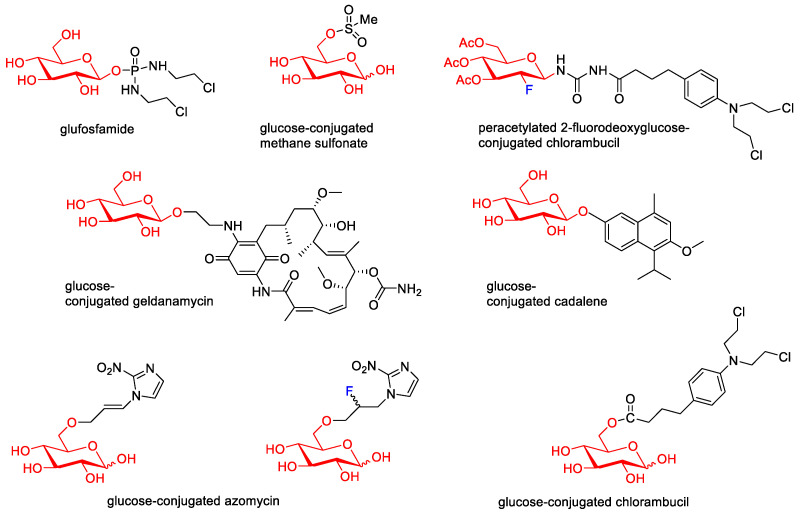
Representative anticancer drug glycoconjugates.

Then, various anticancer drug glycoconjugates targeted to GLUT or ASPGR were developed, based on cytotoxic molecules, such as chlorambucil (CLB) [65], azomycin [66], doxorubicin (adriamycin) [67], and paclitaxel [68,69]. Glycoconjugated prodrugs reported to date consist of a known anticancer drug directly linked via a glycosidic bond to a sugar unit or, as it is in most cases, a known anticancer drug joined to a sugar unit via linkers (esters, amides, ureas, and succinic acids, Figure 2). Commonly used sugar moieties include D-glucose, glucuronic acid, and D-galactose, as well as, to a lesser extent, L-rhamnose and D-xylose.

Numerous troublesome side effects observed for doxorubicin therapy attracted the attention of scientists and contributed to the development of methods to increase its efficacy and, above all, reduce toxicity. Cao et al. designed the molecular hybrid of doxorubicin and 2-amino-2-deoxy-D-glucose, linked via a succinic linker. The 2DG-SA-DOX prodrug, targeting cancer cells by GLUT1, showed higher activity in cancer cell lines (MCF-7 and MDA-MB-231) and lower toxicity to normal cells than DOX, as well as lower organ toxicity [67].

Galactose residues could be specifically recognized by the asialoglycoprotein receptor (ASGPR), which is highly expressed in liver tissues. To explore the possibility of using galactosylated compounds in cancer-targeted therapy, doxorubicin was covalently conjugated with Gal to form a prodrug (Gal-DOX1). The antitumor efficacy of Gal-DOX1 in vitro was assessed in HepG2, MCF-7, and L02 cell lines. The cell viability of tested tumor cells incubated with DOX was higher than that of Gal-DOX1. At the highest dose of 10 μg/mL, the proliferation inhibition of the Gal-DOX1 was 54.3% of total cells, which is much higher than that of DOX (40.1%). Normal cells L02 with lower ASGPR1 level showed high cell viability, suggesting the low cytotoxicity of Gal-DOX1 in cells with low ASGPR1 low expression cells [70].

**Figure 3:**
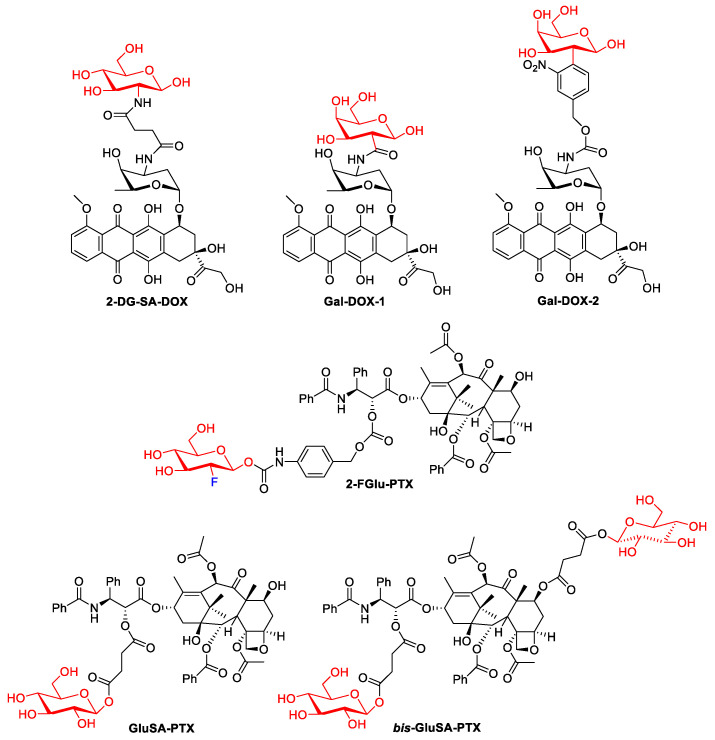
Representative DOX glycoconjugates and PTX glycoconjugates.

In 2018, a theranostic prodrug containing doxorubicin and a galactose moiety connected via a linker (see Figure 3) was used to target asialoglycoprotein receptors [71]. Activation of this prodrug (Gal-DOX2), by β-galactosidases in the colon, releases the parent drug and thus induces a fluorescence phenomenon that allows the monitoring of both the location and site of action of the drug. The imaging properties and therapeutic efficacy of Gal-DOX2 have been demonstrated both in vitro and in vivo in colorectal cancer models (HT-29 and HepG2). The cytotoxicity assays used showed that Gal-DOX2 exhibited a threefold more potent therapeutic effect in HT-29 cells than the HeLa cells. An additional advantage of this glycoconjugate with a simple structure, from a pharmacoeconomic point of view, is its ‘synthetic availability’. In comparison, doxorubicin conjugates with fatty acids, such as α-linolenic acid (LNA) and docosahexaenoic acid (DHA), generated by amide and ester linkages, and as single or double modifications (DOX-monoLNA, DOX-monoDHA, DOX-diLNA, DOX-diDHA) showed lower cytotoxicity against the tested cancer cell lines (SW480, SW620, and PC-3) compared to DOX but higher selectivity than DOX [72].

In 2020, Meng et al. showed that the introduction of fluorodeoxyglucose (2F-Glu) or deoxyglucose in the paclitaxel skeleton significantly improves its solubility (PTX: 0.01 mg/mL, prodrug 2-FGlu-PTX: 0.48 mg/mL, Glu-PTX: 0.87 mg/mL). Glycoconjugate 2-FGlu-PTX exhibited sustained release with less than 50% hydrolysis detected after 12 h, while the non-fluorinated glucose conjugate was unstable (spontaneous release of paclitaxel was observed). 2F-Glu-paclitaxel showed increased cytotoxicity and selectivity (compared to PXL and Glu-PXL) to certain cancer cells (HepG2, NCI-H460, MCF-7) [73].

In another study, glycoconjugates of paclitaxel with single or double glucose moieties attached by succinate linkers were reported [74]. Both the C-2′-single glycosylated paclitaxel (GluSA-PTX) and the double glycosylated paclitaxel (*bis*-GluSA-PTX) conjugates showed effective cytotoxicity against breast cancer cells and improved hydro solubility compared to the parent drug. The improved solubility obtained is very important because it allows the elimination of the toxic surfactant *Cremophor EL* (polyethoxylated castor oil), which has so far been used as a carrier in paclitaxel treatments.

**Figure 4:**
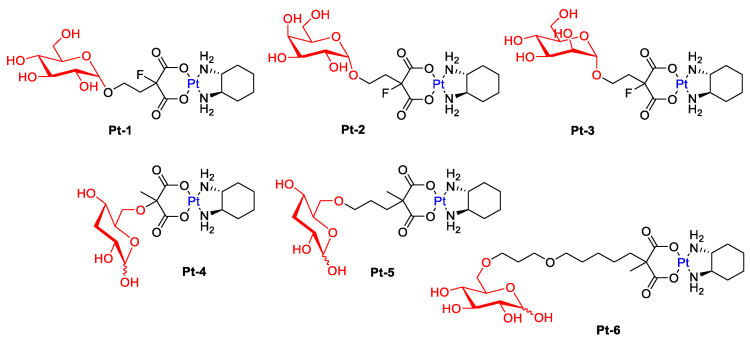
Representative Pt-complex glycoconjugates (conjugated via C-1: Pt-1–Pt-3 and conjugated via C-6: Pt-4–Pt-6).

The study presented by Han’s group showed that oxaliplatin modified with various sugar units, such as glucose, mannose, or galactose (Figure 4), has greater anticancer activity compared to the parent drug [75]. The tests suggest that the cytotoxicity of water-soluble platinum(II) complexes (Pt-1, Pt-2, Pt-3) is related to glucose transporters.

Patra et al. hypothesized that a modification at the C6 position of D-glucose should also have a positive effect on receptor binding (GLUT1) [76]. Furthermore, the Pt-6 complex (Figure 4) has been proven in vitro to preferentially kill cancer cells while exhibiting reduced accumulation and low toxicity to normal cells. The translocation efficiency and subsequent cellular accumulation decreased with increasing linker length.

**Figure 5:**
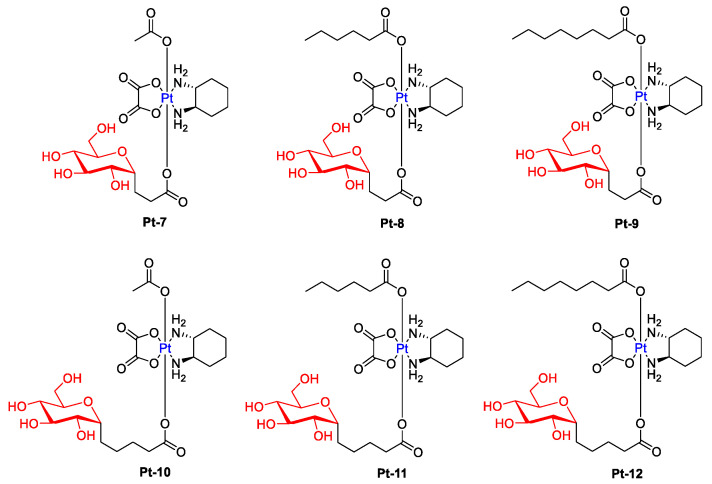
Chemical structures of glycosylated Pt(IV) complexes with different linkers (Pt-7–Pt-12).

An interesting example of glycoconjugated drugs is six glycosylated Pt(IV) compounds, presented in Figure 5, whose cytotoxicity was evaluated against five human cancer cell lines (Hela, HepG2, MCF-7, A549, and A549R) and normal human liver compared with cisplatin and oxaliplatin [77]. These complexes showed a higher level of apoptosis-inducing and lower cytotoxicity to normal LO2 cells than cisplatin and oxaliplatin while maintaining antitumor activity (Selectivity Index for Pt-10: 14.14, Pt-11: 24.1, Pt-12: 10.9, Cisplatin: 0.88, Oxaliplatin: 0.77). Additionally, the effect of alkyl ligands on cytotoxicity was greater than that of the ligand between the glycoligand and the Pt nucleus (Pt-9 had higher cytotoxic effects than Pt-10).

Vaidya and Patra presented an overview of glycoconjugation as an attractive strategy to impart selectivity and improve pharmacokinetics of platinum-based anticancer agents [78]. An interesting critical review article on the topic of progress (over the past decade 2010–2020) in glycoconjugation of anticancer drugs describes Fu et al. [79] and the Martin group [80]. The review works testify to the multitude of works carried out on improving the properties of platinum-based drugs using the glycoconjugation strategy and, consequently, the great interest in this subject. We summarize selected examples of anticancer drug glycoconjugates (ADG) in Table 1.

Methotrexate (MTX, amethopterin), with both antiproliferative and anti-inflammatory properties, was introduced as a drug in 1953. It is used as a cytostatic drug in the treatment of cancers such as acute myeloid leukemia, acute lymphoblastic leukemia, breast, ovarian, lung, prostate, bladder, osteosarcomas, and solid tumors of the head and neck. It is also used in the treatment of autoimmune diseases such as psoriasis and rheumatoid arthritis [81]. MTX, as a folic acid antagonist, inhibits the activity of dihydrofolate reductase (DHFR), which catalyzes the conversion of dihydrofolate to tetrahydrofolate, which in turn is necessary for the synthesis of nucleotide bases. As a result, it leads to DNA and RNA synthesis disorders, inhibition of cell division, and, ultimately, cell death. The target of MTX action are all rapidly proliferating cells, including cancer cells, bone marrow, fetal cells, oral and intestinal mucosa, and bladder cells. MTX enters cells through protein folate transporters (RFC1 or FBP), and in higher concentrations, after saturation of transporters, through passive diffusion [82]. Although this medicine in the treatment of autoimmune diseases used in small doses is relatively safe, high doses used in oncological therapies cause very high systemic toxicity. Methotrexate, as a drug that acts nonspecifically on all body cells, can cause strong side effects, including, among others, gastrointestinal complaints, inflammation of the skin and blood vessels, kidney and liver failure, lung and intestinal diseases, and damage to the bone marrow and mucous membranes [83]. To avoid the undesirable consequences of the use of MTX, it is advisable to develop new strategies that will allow the selective delivery of methotrexate to the targeted sites, thus limiting side effects. Recently, many selective drug delivery systems have been developed [84]. MTX prodrugs activated under tumor microenvironment conditions were also obtained. An interesting example is glutathione-activated conjugate-based theranostic prodrug (Cy-SS-MTX) based on heptamethine cyanine (Cy) conjugated to MTX via a disulfide bond. This prodrug was activated by a high GSH level in the tumor, leading to a change in the optical properties of Cy group. This made it possible to track the activation of the administered prodrug under different excitation wavelengths. The obtained prodrug was tested both in vitro on four cell lines (MCF-7, SKOV-3, A549, and MCF-10A) and in vivo (mice bearing MCF-7 tumor). Based on the conducted research, it was found that prodrug improved anti-tumor efficiency of MTX and significantly reduced its toxicity to healthy cells [85]. However, there are few reports on the modification of the MTX molecule by its glycoconjugation. In 2001, the synthesis of conjugates was described, in which the carboxyl groups of the MTX molecule were connected to the anomeric position of per-*O*-acetylated-β-D-glucopyranosylamine through a linker made of lipoamino acids with three different alkyl chain lengths (LAAG-MTX). The cytotoxicity of the compound obtained against the lymphoblastic leukemia cell line (CCRF-CEM) was tested. Unfortunately, in vitro, this glycoconjugate turned out to be less active than the MTX derivative conjugated only with lipoamino acid [86].

**Figure 6:**
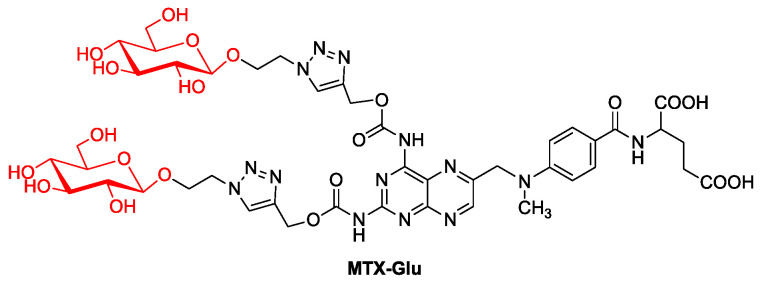
MTX glycoconjugate.

Perhaps the failure of the first described MTX glycoconjugate made another MTX glycoconjugate described only in 2021 (Figure 6, MTX-Glu). It was designed on the assumption of the best affinity for GLUT receptors. Therefore, attached D-glucose molecules are not protected by protecting groups and have a β-configuration of the substituent at the anomeric carbon, as this orientation is preferred by GLUT proteins [87]. The 1,2,3-triazole ring in the linker between the sugar part and the drug increases the affinity of the compound for the transporter due to the possibility of hydrogen bonding. In turn, the linker structure with a glycosidic bond on the sugar side and a carbamate bond on the MTX side is selected to ensure the stability of the molecule in the extracellular environment and the possibility of chemoenzymatic degradation inside the cell [88]. The results of activity assays of the obtained MTX-Glu glycoconjugate allowed us to conclude that it has a strong cytotoxic effect in the in vitro environment against a wide panel of cancer cell lines, similar to the activity of unmodified MTX. This has also been confirmed in in vivo studies targeting breast cancer in mice. At the same time, the MTX conjugate showed low toxicity to noncancer cells, which significantly improved the selectivity of the drug. Additionally, the uptake of glycoconjugate by tumor cells and its accumulation in the intracellular compartment are significantly more efficient compared to MTX, which may indicate facilitated transport of the glycoconjugate by targeting GLUT1 transporters, its cellular distribution and intracellular release of the active molecule [89,90].

**Table 1:** Representative anticancer drug glycoconjugates.

Drug	Conjugated Sugar	Type of Anticancer Activity Studies; Transportation Mode	Activity Compared to Glycone/Properties	Ref.
Ifosfamide	D-Glucose	Alkylating agent	Glufosfamide - less toxic in vitro - antitumor activity (in vitro and in vivo) higher than the parent aglycone - the first human clinical trial to test glufosfamide (1997, Europe) [63] - a phase-II study of glufosfamide against pancreatic cancer (2010) [91]	[62]
Doxorubicin (DOX, ADM)	2-amino-2-deoxy-D-glucose and succinic acid	Antitumor antibiotic GLUTs mediated	2DG–SA–DOX - higher anticancer activity than DOX against cell lines - (MCF-7, HepG2; in vitro and in vivo) - induces a higher level of apoptosis - highly specific against cancer cells - no effect on normal cells	[67]
Doxorubicin (DOX, ADM)	Galactose	Antitumor antibiotic	Gal-DOX1 - higher anticancer activity than DOX against cell lines (MCF-7, HepG2) - a significant reduction in tumor size compared to DOX therapy (in vivo experiments) - targeting cancer cells (for the group treated with Gal-DOX1 survival was 100%, for the group treated with DOX—50%)	[70]
Doxorubicin (DOX, ADM)	Galactose	ASPG mediated	Gal-DOX2 - theranostic prodrug activation by β-galactosidase enzymes in the colon induces a fluorescence phenomenon that allows monitoring of the location and site of action of the drug - cytotoxicity assays in vitro and in vivo in colorectal cancer models (HT-29, HepG2) exhibited a 3-fold more potent therapeutic effect in HT-29 cells than the HeLa cells	[71]
Chlorambucil (CLB)	Amino derivatives of glucose, mannose, galactose, xylose, lyxose, D-threoside	Alkylating and DNA-complexing agent	D-threoside-CLB - the most active chlorambucil neoglycoside among the compounds tested - 8-fold higher efficacy in general - HT29 (12-fold), HCT15 (15-fold) improved activities targeting cancer cell lines (from colorectal adenocarcinomas) over the parent drug	[92]
Chlorambucil (CLB)	Peracetylated 2-fluorodeoxyglucose		FDG-CLB - this preclinical study in 2 murine xenograft models showed high antitumor activity, on the basis of LCK values higher than 1.5 times - MCF-7 (human fibroblasts, 25-fold more active)	[93]
Paclitaxel (PTX)	Glucose	Mitotic inhibitor	Glu-PTX - increase in solubility in water - induces chromosome condensation and tubulin aggregation	[68,69,94]
Paclitaxel (PTX)	Glucose		2FGlu-PTX/PTX - HepG2, IC_50_: 0.89/1.58 µM - NCI-H460, IC_50_: 1.85/2.37 µM - MCF-7, IC_50_: 0.002/0.005 µM - HUVEC, IC_50_: 10.25/7.16 µM	[73]
Paclitaxel (PTX)	Glucose		a single (GluSA-PTX) and double (*bis*-GluSA-PTX) - show effective cytotoxicity against breast cancer cells - improve hydrosolubility than PTX	[74]
Azomycin	Glucose	GLUTs-mediated	Glucoazomycins - are radiosensitizers (in vitro test) - competitively inhibit glucose uptake (in vitro test)	[66]
Geldanamycin (GA)	Glucose	HSP90 inhibitor	Glu-GA - IC_50_: 70.2–380.9 nM in various cancer cells (SW620, HT29, MCF-7, K562) - by-glucosidase activation inside of the tumor cells (inhibition using β-glucosidase specific inhibitor)	[95]
Geldanamycin (GA)	Galactose Lactose	HSP90 inhibitor	Gal-GA and Lac-GA - 3–40-fold improve activities against SW620, HT29, MCF-7, and K562 when incubated with β-galactosidase in the cells	[95]
Emodin (EM)	D-rhamnose	Tyrosine kinase inhibitor	Rha-EM - is 10-fold more effective than EM in inhibited cell proliferation - shows strong anticancer activity against a human cancer cell lines (HepG2, K562, Hela, SGC-790, A594, OVCAR-3)	[96]
Platinum	Glucose	GLUTs mediated	Glucose-conjugated Pt(IV) complexes - show enhanced cytotoxicity to five human cancer cell lines (MCF-7, Hela, HepG-2, A549,A549R) compared to cisplatin and oxaliplatin due to the transport-mediated effect of GLUTs	[77]
Oxaliplatin	Glucose, Mannose Galactose	GLUTs mediated	- improvement of cytotoxicity	[75]

aASGP-R: asialoglycoprotein receptor (ASGP-R), GLUT1: glucose transporter 1, GLUT5: glucose transporter 5, PIC: polyion complex.

### 2.2. Glycoconjugates of Biological Active Compounds

**Figure 7:**
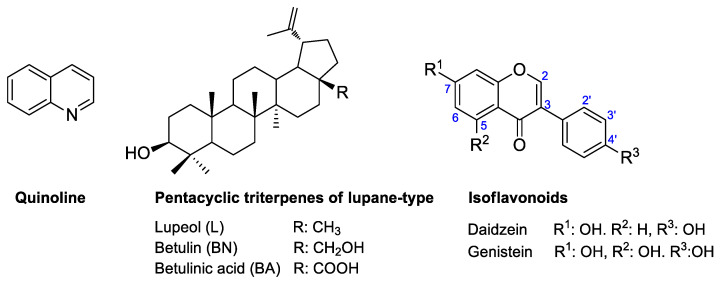
Selected natural bioactive compounds.

Natural compounds often possess promising therapeutic properties, which causes are of constant interest to scientists as potential drugs or prodrugs for the treatment of many diseases [97]. Many of them, including quinoline derivatives, pentacyclic triterpenoids (PT), or flavonoids, such as genistein, daidzein or quercetin (Figure 7), exhibit a broad spectrum of various bioactivities, especially antitumor, antiviral, or antibacterial activity [98,99,100,101,102,103]. However, their use as therapeutic agents is limited because of low bioavailability (the poor aqueous solubility), low systemic circulation half-time, unsatisfactory selectivity, and insufficient intracellular accumulation. Usually, the hydrophobic nature of the native skeleton of a natural compound hinders its ability to reach the target in vivo and obtain the desired therapeutic effect in acceptable therapeutic doses. Several chemical modifications of parent structures have been described in the literature to improve their physicochemical and pharmacokinetic properties. An interesting solution is the synthesis of low-molecular-weight hybrids and conjugates, such as glycoconjugated derivatives. Here, we present a review of research, in which the influence of glycoconjugation on the properties and action of selected natural biologically active compounds was assessed.

Quinoline and its derivatives play an important role as key structural units of many natural compounds and important drugs and are useful building blocks for new biologically active compounds [104,105,106]. An example of a quinoline derivative, interesting from the point of view of a wide spectrum of biological activities and also easy to modify structurally, is 8-hydroxyquinoline (8-HQ). Its skeleton is a privileged structure used to design compounds with a variety of therapeutic effects, such as clioquinol, intestopane, nitroxoline, or chloroquinaldol. There are many reports on their antibacterial, antifungal, antiprotozoal, antineurodegenerative, as well as disinfectant and antiseptic properties [107,108,109]. In recent years, for compounds containing the 8-HQ skeleton in their structure, there has been an increase in interest in the anticancer activity, resulting from their ability to chelate copper ions necessary in the carcinogenesis process [110,111]. Quinoline derivatives show antiproliferative activity in a wide spectrum of different cancers, as they can interfere with many different signaling and enzymatic pathways [105,106]. Additionally, due to the planar structure of the quinoline backbone, they can also intercalate between DNA base pairs, leading to conformational changes and DNA strand breaks [112]. Despite such a variety of effects, 8-HQ derivatives have only limited use in anticancer therapies. One of the reasons is the lack of specificity for pathological cells, whose consequence is the ability to interact with all ions in the body, as well as various proteins and enzymes encountered after administration to the body. As a consequence of nonselective chelation of transition metal ions, which are needed not only for cancer cells but are also cofactors necessary for the proper running of many important cellular processes essential for the proper functioning of the entire organism.; the balance of metal ions in healthy tissues may be disturbed, and thus it is important to maintain their homeostasis [113,114]. An additional difficulty in the use of 8-HQ derivatives in the therapy of numerous diseases is their toxicity and poor bioavailability. Therefore, its derivatization is important, and one of the methods of such derivatization is glycoconjugation, which aims to take advantage of the increased glucose demand of tumor cells to target 8-HQ derivatives directly to the tumor. The use of 8-HQ connections with sugars has been described in several papers. These were connections by the direct formation of a glycosidic bond between the sugar and the 8-HQ derivative, as well as by the use of various linkers. In addition, both the sugar anomeric position and other positions, particularly the C-6 position, can be used to conjugation.

**Figure 8:**
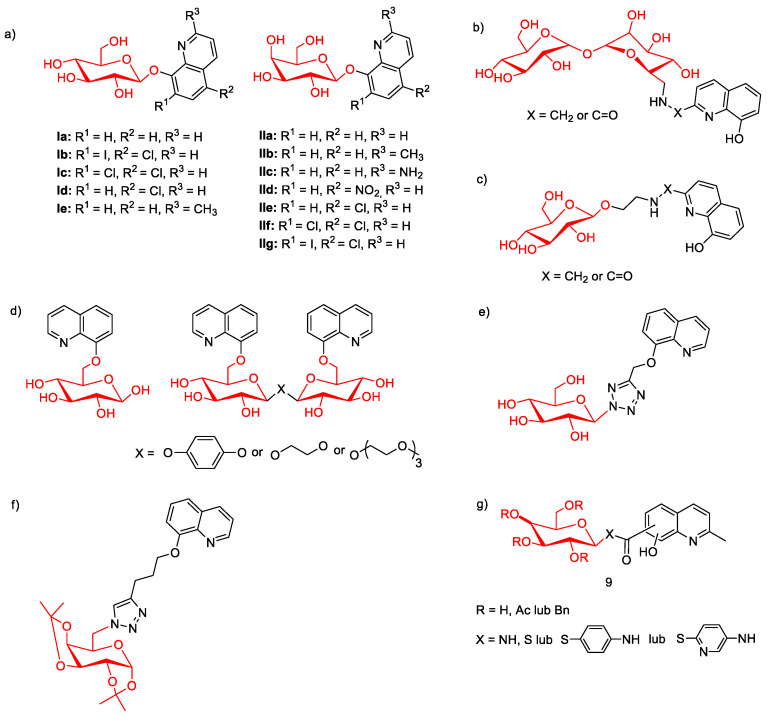
8-Hydroxyquinoline glycoconjugates (**a**–**g**).

A library of 8-HQ *O*-glycosides, containing both D-glucose and D-galactose residues (Figure 8a), was obtained, and cytotoxicity was tested by G. Vecchio’s team. Studies on the antiproliferative activity of these compounds were carried out in cells of various types of cancer, both without and with the addition of Cu^2+^ ions. The results of these studies showed that, in most cases, the average antiproliferative activity of the glycoconjugates without the addition of Cu^2+^ ions was lower than its parent compounds. The addition of Cu^2+^ ions to the system caused an increase in the activity of glycoconjugates to the level of activity of the initial 8-HQ derivatives. The authors suggest that the attached sugar unit temporarily masks the chelating functions of these glycoconjugates until the hydrolytic release of the active aglycone catalyzed by intracellular β-glycosidases [115,116,117]. The same team described the biological properties of 8-HQ glycoconjugates in which trehalose or D-glucose was linked to 8-HQ via a linker (Figure 8b,c). In the case of these compounds, a linker attached to the C-2 position of the quinoline derivative was used to connect the sugar to 8-HQ. This modification improved the solubility of the obtained derivatives in water, which made it possible to perform tests in physiological conditions, which showed their ability to form bonds with Cu^2+^ and Zn^2+^ ions. Unfortunately, these glycoconjugates did not show antiproliferative activity against tested cancer cell lines (A2780, A549, and SHY5Y); however, high antioxidant and anti-aggregation activity was demonstrated for the trehalose derivatives [118]. Another research team obtained quinoline glycoconjugates by coupling 8-HQ to the 6-OH group of D-glucose and symmetrically linking two sugar molecules through various linkers, such as quinol, glycol, or triethylene glycol, substituted at the anomeric position of both sugar molecules (Figure 8d). The planar aromatic system present in these molecules allows them to slip between adjacent DNA base pairs, allowing these compounds to act as intercalating agents interacting with DNA. Glycoconjugates and their acetylated counterparts moderately inhibited the growth of MDA-231 human breast cancer cells. The best activity was described for the glycoconjugate with an aromatic ring in the linker [119].

There are also reports in the literature on the antiproliferative activity of 8-HQ derivative glycoconjugates containing an additional heterocyclic fragment in the structure. In 2010, the synthesis of an 8-HQ derivative glycoconjugate containing a tetrazole linker and a D-glucose unit in its structure (Figure 8e) was described, characterized by better water solubility and bioavailability than 8-HQ. This compound showed high cytotoxic activity against breast cancer cells (MCF-7), comparable to that of cisplatin. The anticancer effect of this molecule has been shown to be associated with the generation of reactive oxygen species (ROS), the high level of which leads to cell death [120]. In 2014, a quinoline glycoconjugate with a 1,2,3-triazole fragment was described in the linker between 8-HQ and galactose, which was obtained in the 1,3-dipolar azide-alkyne cycloaddition reaction (CuAAC) between 8-*O*-alkyne quinoline derivative and 6-azido-D-galactose (Figure 8f). The compound was tested in vitro for its antiproliferative activity against various types of human tumor cells. This derivative showed the highest cytotoxicity and selectivity for ovarian cancer cells (OVCAR-03), exceeding the activity of DOX as a reference drug. It is worth mentioning that the same work also describes eleven other 8-hydroxyquinoline conjugates obtained by CuAAC reactions between 8-*O*-alkyne quinoline derivatives and various aromatic azides. Physicochemical parameters were calculated for all obtained compounds based on structure–activity relationship (SAR) studies, and the obtained SAR results appeared promising. Authors, based on the results of the calculations, indicate the important role of the location of the HOMO and LUMO orbitals in the studied compounds for their activity toward prostate cancer cells. All these compounds were also tested in vitro for their antiproliferative activity against various types of human tumor cells. Among the aromatic 8-hydroxyquinoline conjugates, the chlorinated ones showed the best overall activity [121]. Other glycoconjugate derivatives of hydroxyquinoline carboxylic acids (2-methyl-8-hydroxy-7-carboxyquinoline or 2-methyl-5-hydroxy-6-carboxyquinoline) were obtained through the connection of sugar derivatives with a quinoline carboxyl group, leaving a free quinoline hydroxyl group, which is key in the ion chelation process (Figure 8g). The obtained glycoconjugates were tested for antitumor activity against the HCT 116 cell line. It has been observed that a significant improvement in biological activity occurs in the presence of a heteroaromatic linker between the sugar and the quinoline fragment. Compounds with an additional pyridine ring in the linker showed greater than 100 times more cytotoxicity than the original quinoline derivatives. The obtained glycoconjugates showed increased activity in the presence of a high concentration of Cu^2+^ ions. Assessing the possible mechanism of action of the tested compounds, it was deduced that the cytotoxicity was associated with the generation of ROS and DNA intercalation. Unfortunately, despite the presence of a sugar subunit, these compounds were not transported by GLUT proteins, and their activity did not depend directly on glucose metabolism [122].

**Figure 9:**
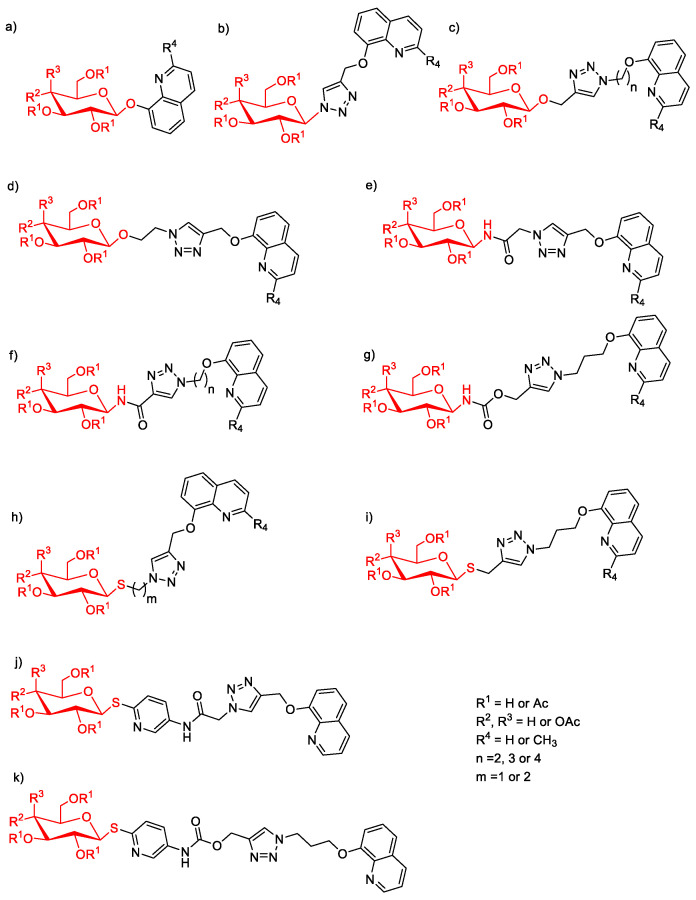
8-Hydroxyquinoline glycoconjugates containing a 1,2,3-triazole ring in the linker structure (**a**–**k**).

**Figure 10:**
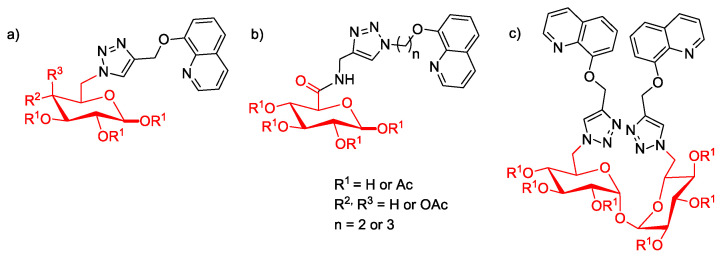
8-Hydroxyquinoline glycoconjugates containing sugar functionalized at C-6 position (**a**–**c**).

In the years 2019–2021, several articles were published describing the synthesis of quinoline glycoconjugates, in which derivatives of D-glucose, D-galactose, glucuronic acid or trehalose were connected with 8-hydroxyquinoline or 8-hydroxyquinaldine directly through the *O*-glycosidic linkage or via a linker containing 1,2,3-triazole ring. Both the anomeric position (Figure 9) and the C-6 position of the sugars were used to obtain these glycoconjugates (Figure 10). In the case of glycoconjugates in which the anomeric position of sugars was used for conjugations, the described compounds contained different atoms attached to the sugar anomeric position, i.e., oxygen, nitrogen, or sulfur. This made it possible to check whether the nature of the atom at this position affects the activity of the obtained glycoconjugates.

All of these glycoconjugates have been studied to evaluate their potential in cancer treatment using cell lines in which overexpression of the glucose and galactose transporters was observed: HeLa, HCT 116, MCF-7, U-251, Hs683, PANC-1, and AsPC-1. For the most active compounds, to assess their selectivity, cytotoxicity tests against neonatal human dermal fibroblasts (NHDF-Neo) cells were performed [123,124,125,126]. The results of the studies showed that the 8-HQ *O*-glycosides did not show any cytotoxicity against the tested cell lines. Only linking sugar with 8-HQ through a linker containing a 1,2,3-triazole ring allowed obtaining glycoconjugates with anticancer activity. The fact that the glycoconjugates containing the unprotected sugar fragment showed negligible cytotoxicity indicated there is hardly anything to say about their affinity for the GLUT transporters. Their definitely more cytotoxic counterparts with a sugar fragment protected by acetyl groups because of greater lipophilicity probably entered the cells through passive transport. This is also confirmed by the observed cytotoxicity of the protected glycoconjugates against healthy cells. The exception was the 8-HQ glycoconjugate and a protected D-glucose derivative (Figure 9b), for which the IC_50_ value for MCF-7 cells was 4.12 µM, while the IC_50_ value for NHDF-Neo cells was 31.91 µM [123]. Modifications in the structure of quinoline glycoconjugates, related to the extension of the alkyl chain between the 1,2,3-triazole ring and the quinoline or sugar moiety, as well as the introduction of an additional amide, carbamate, or heteroaromatic ring to the linker structure, as well as the type of atom attached to the anomeric position of the sugar and the spatial orientation of the 1,2,3-triazole ring in the linker did not significantly improve their antitumor activity (Figure 9c–k). It could only be noticed that the introduction of sulfur into the anomeric position of the attached sugar improves the hydrolytic stability of the obtained glycoconjugates, whilst the introduction of the amide moiety and the additional heteroaromatic pyridine moiety into the structure of the linker slightly increases the cytotoxicity of the glycoconjugates but does not improve their selectivity [124,125].

Glycoconjugates in which 8-HQ derivatives were conjugated at the C-6 position of the sugar unit (Figure 10) were also described. These derivatives turned out to be more cytotoxic and, at the same time, more selective than the analogous glycoconjugates formed by the C-1 position of D-glucose. The selectivity index of the unprotected D-glucose glycoconjugate (Figure 10a) determined for HCT 116 cells was 14.21. Moreover, unlike the glycoconjugates obtained through sugar anomeric functionalization, the new series of glycoconjugates containing the unprotected glucose moiety are significantly more selective for tumor cells compared to the derivatives containing the protected sugar moiety. The results of the cytotoxicity test carried out in the presence of a GLUT1 transporter inhibitor suggest that they may be involved in the uptake of a new series of glycoconjugates. These glycoconjugates showed pro-apoptotic properties without significantly affecting changes in cell cycle distribution and were able to reduce the clonogenic potential of tumor cells and inhibit cell migration and DNA intercalation [126].

Another group of compounds that are interesting from the point of view of anticancer activity are triterpenoid saponins. A detailed description of the biological and pharmacological effects of most of the known synthetic triterpenoid saponins and steroid saponins was presented by Juang’s team in 2020 [127]. Our discussion focuses on the development of pentacyclic lupane-type triterpene glycoconjugates and their anticancer activity.

For several decades, naturally occurring lupane-type pentacyclic triterpenes have been widely researched in terms of the search for new potential therapeutic agents [98,128]. Their native scaffolds are the basis for designing new drugs. Above all, betulinic acid (BA, 3β, hydroxy-lup-20(29)-en-28-oic acid) and betulin (BN, lup-20(29)-ene-3,28-diol) attract the attention of scientists as each possesses a multidirectional spectrum of biological properties, such as anti-HIV, antitumor, anti-inflammatory, antimalarial, immunomodulatory, and hepatoprotective [129,130]. Betulinic acid has gained popularity due to its anticancer properties against many tumors. In addition, BA is characterized by high selectivity toward cancer cells and a favorable safety profile. Several mechanisms of its action are postulated, for example, arrest of the cell cycle, induction of apoptosis, immune regulation, reversal of multidrug resistance (MDR), or induction of autophagy [131]. On the other hand, betulin is a readily available triterpenoid found in the plant kingdom. An extremely rich source of betulin is birch bark, for example, *Betula* sp. (the BN content is up to 34% and BA only 0.3% of dry weight) [130].

**Figure 11:**
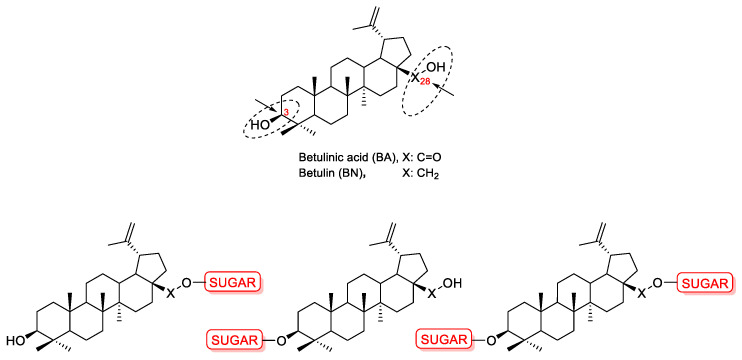
Chemical modifications of the parent pentacyclic lupane-type triterpene via connection with the sugar moiety at position C-28 and/or C-3 position.

These natural bioactive compounds with a high safety profile are considered interesting materials for making a variety of structural modifications. They have high synthetic potential because their parent skeleton is enriched in easily transformable functional groups (e.g., BA: C3-OH, C28-COOH, and BN: C3–OH, C28–OH). One strategy that makes it possible to improve pharmacokinetic properties (e.g., solubility and bioavailability, selectivity in targeting drugs for a specific purpose) is to conjugate the native triterpenoid backbone with a sugar moiety, as shown in Figure 11.

There have been many reports on glycoconjugate triterpenoids, called saponins, of which the selected are presented in Table 2.

At first, the anticancer activity of a series of 3β-*O*-monoglycoconjugates derived from betulinic acid (BA) and its methyl ester, betulin (BN), lupeol (L), and allobetulin (AlloBN) based on six different natural sugar residues (D-glucose, L-rhamnose, D-arabinose, D-galactose, D-mannose, and D-xylose) was evaluated in vitro [132,133]. These studies of structure–activity relationship showed that the introduction of a sugar unit into the parent skeleton has a positive effect on the pharmacological properties and in all tested cases improves hydrosolubility. However, cytotoxicity is strongly dependent on the structure of glycone and aglycone (Table 2, Entry 1–5). The best research results were obtained for the 3β-*O*-L-rhamnopyranoside derivative of betulinic acid because it showed that lung, colorectal adenocarcinoma, and mouse melanoma cancer cell lines are 8- to 12-fold more sensitive to this 3β-*O*-Rha-glycoconjugate betulinic acid (IC_50_: 2.6–3.9 µM) than the healthy cells (IC_50_: 31 µM, Table 2, Entry 1). Furthermore, the addition of a sugar moiety (D-Glu, L-Rha, D-Ara) at the C-3 or C-28 position of BN resulted in a loss of cytotoxicity of the native skeleton (against A549, DLD-1, and B16-F1 cell lines), whereas, the 3β-*O*-D-glucosidation of lupeol improve his activity by 7- to 12-fold (IC_50:_ 14.0–15.0 μM, Table 2, Entry 4).

Next, it was first reported that the synthesis of the betulinic acid 28-O-β-D-glucuronide was carried out with success in a stereoselective and efficient manner under phase-transfer conditions [134]. Admittedly, this new prodrug (Table 2, Entry 6) was not cytotoxic against the tested cell lines. However, it underwent enzymatic hydrolysis when treated with β-D-glucuronidase, an enzyme more common in tumor tissue than in healthy tissue. In vitro, it released 75% betulinic acid after 24 h [134]. 28-*O*-β-D-glucuronide betulinic acid is, therefore, a promising anticancer agent, which in the future, may be used in prodrug therapy because it is non-cytotoxic, non-hemolytic, more soluble in water than BA, and quite stable in phosphate buffer.

Gauthier et al. also published the synthesis of naturally occurring saponin 28-*O*-β-D-glucopyranosylbetulinic acid 3β-*O*-α-L-arabinopyranoside and seven glycoconjugates containing two sugar units starting from BN or BA [135]. The preliminary cytotoxicity evaluation of the betulin analog, which carries R-L-rhamnopyranoside moieties at the positions C-3 and C-28 against A549, DLD-1, MCF-7, and PC-3 human cancer cell lines, indicated it was a potent cytotoxic agent (IC_50_: 1.8–1.9 μM, (Table 2, Entry 7). The biological activity of naturally occurring saponins and many synthetic glycoconjugates of pentacyclic lupane-type triterpenoid was described in a review [136].

In another study [137], researchers prepared a series of five glycoconjugates of the lupane- and germanicane-type bearing a chacotrioside moiety at the C-3 position, using a stepwise glycosylation strategy and evaluated for both their cytotoxic and hemolytic activities. This study showed that the chacotriose moiety increases the hemolytic activity of the less polar triterpenoids, that is, lupeol, allobetulin, and 28-oxoallobetulin. Additionally, allobetulin chacotrioside (Table 2, Entry 8), which are betulin rearrangement products, proved to be an interesting compound for further in vivo studies since it is weakly hemolytic (HD_50_: 90 ± 9 µmol L^−1^) and exhibited a good cytotoxicity profile against cell lines derived from the most prevalent human cancer (A549, DLD-1, MCF-7, and PC-3). Unfortunately, it was also cytotoxic to the human normal fibroblast cell line (WS1).

Cmoch et al. designed and prepared a series of glycoconjugates from three lupane-type triterpenes (L, BA, and BN) modified with mono-, di- or trimannosyl residues; they could provide a convenient means of delivering drugs to certain human cells through interactions with mannose receptors [138]. Although many of these glycosides exhibited only moderate to low cytotoxicity against tested cancer cell lines, several monomannopyranosidic derivatives (Table 2, Entries 9–10) exhibited higher cytotoxicity than the precursor (di- or trisaccharide analogs were inactive) [138].

The hemolysis of red blood cells that induces toxicity in animals and humans is a major drawback for the clinical development of triterpenoid glycoconjugates as antitumor agents. Gauthier has shown that lupane-type glycoconjugates do not exhibit any hemolytic activity at the maximum concentration tested (100 µM) independent of the nature of the sugar moieties [139]. The change in cytotoxicity to many tumor cell lines was observed as a result of BA derivatization at the C-3 position by α- and β-anomers of D-glucopyranose [139]. The promising candidate for biological evaluation is the B10 betulin derivative (Table 2, Entry 11), synthesized by Kommera et al. [140]. Gonzalez et al. postulated that B10 simultaneously induces autophagy and inhibits autophagic flux, which can turn autophagy into a mechanism of cell death [141]. This means that apoptotic and nonapoptotic cell death coexists during the use of this glycoconjugate, which is important, especially in the case of apoptosis-resistant cancers. Sylla synthesized new glycoconjugates of betulinic acid with mono-, di-, tri-, and tetra-α-L-rhamnose moieties in high yields with complete control of stereoselectivity [142]. It was found that the presence of one or two units of sugar positively modulates anticancer activity. The betulinic acid glycoconjugate with a 3-*O*-α-L-rhamnopyranosyl-(1/4)-α-L-rhamnopyranosyl residue appears to be a potent cytotoxic agent against human colorectal adenocarcinoma cells without damaging healthy cells (selectivity ratio > 20, Table 2, Entry 12).

An interesting group of glycoconjugates are betulin analogs modified at positions C-3 and C-28 with sugar moiety, presented by Korda’s team [143]. Cytotoxicity of all compounds was tested in vitro for a series of cancer cell lines (CEM, MCF-7, HeLa, and G-361), as well as normal human skin BJ fibroblasts. It was shown that the presence of two units of sugar causes a strong increase in cytotoxicity, unfortunately, also in relation to healthy cells. (Table 2, Entry 13).

In several articles, Pakulski et al. described a series of lupane glycoconugates, modified at the C-3 position, including 3-*O*-glycoside, 28-COO-glycoside, 28-COO-thioglycoside, and 28-COO-selenoglycoside [144,145,146]. However, all the compounds tested were either poorly cytotoxic to cancer cells or poorly selective for cancer cells over normal cells.

Mihoub et al. in 2018 prepared and evaluated as anticancer agents a series of polar glycosylated BN derivatives [147]. The most favorable cytotoxicity (IC_50_: 2.9–5.9 µM) against the tested lung cancer cell lines (A549, LLC1, NCI-H522, NCI-H1993, NCI-H2087, NCI-H1755) was shown by rhamnopyranose modified betulin both in the C-3 position as well as C-28 (3,28-*bis*-*L*-RhamBN). At a dose of 50 mg/kg, a significant tumor growth inhibition effect was observed (46% on 18 days). It was reported that 3,28-*bis*-*L*-RhamBN caused reduced ROS production and decreased membrane potential; thus, it can induce apoptotic cell death.

**Figure 12:**
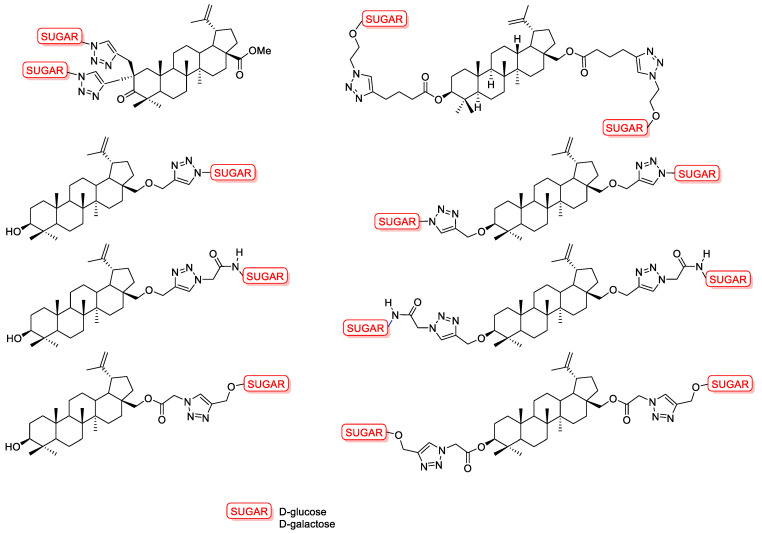
Pentacyclic triterpenes lupane-type glycoconjugates obtained by *Click chemistry*.

The next papers showed that an effective way to synthesize new glycoconjugates based on naturally occurring triterpene type-lupane provides copper-catalyzed 1,3-dipolar azide-alkyne cycloaddition reaction (CuAAC) (Figure 12). Spivak et al. [148] reported the synthesis of BA derivatives (modification at the C-2 position, Glu(OAc)), while Grymel et al. [149] developed a chemoselective method for the synthesis of mono- and disubstituted betulin derivatives containing sugar units (Glu(OAc), Gal(OAc)) attached via different linkers inclusive 1,2,3-triazole ring at the C-3 and/or C-28 position of the parent skeleton. The preliminary cytotoxicity assay (on MCF-7 and HCT 116 cell lines) of the obtained BA and BN glycoconjugates showed that the addition of a sugar unit to the native structure is not significant for biological activity.

Yamansarov et al. described the synthesis and biological evaluation in silico, in vitro, and in vivo of six new glycoconjugates obtained by attaching *N*-acetyl-D-galactosamine fragments (one or two saccharide ligands) to the C-3 or/and C-28 positions of the betulin molecule using triazole as a linker [150]. These molecules demonstrate high affinity for the asialoglycoprotein receptor (ASGPR) of hepatocytes assessed by in silico modeling and surface plasmon resonance tests. In vitro cytotoxicity studies revealed that di-conjugate acts with moderate activity and selectivity against HepG2 hepatocellular carcinoma cells (IC_50_: 25.9 µM, for BN IC_50_: 4.2 µM.; Table 2, Entry 15). Studies of the in vitro cellular uptake and the real-time microdistribution in the murine liver in vivo analog showed its selective internalization into hepatocytes due to the presence of GalNAc ligand in comparison with reference compounds.

Researchers have investigated different biological aspects of triterpene-type lupane, such as new formulations for improving their bioavailability, targeted drug delivery, and designing new derivatives of B and BA with improved therapeutic efficacies. Unfortunately, despite numerous reports on the benefits and therapeutic properties, few clinical trials in the literature described the effects of these compounds on humans.

**Table 2:** Representative glycoconjugates from triterpenoids (lupeol, BN, BA).

Entry	Glycoconjugates	Attached Sugar	Tested Cell Line	Methodology	Best Research Effects IC_50:_ Glycoconjugates/IC_50_ Precursor	Ref.
1.	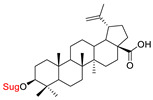	D-Glu, L-Rha D-Ara, D-Gal D-Man D-Xyl	A549 DLD-1 B16-F1 WS1	resazurin reduction test (RTT), in vitro	L-Rha(OH): A549, IC_50_: 2.6/10.3 µM DLD-1, IC_50_: 3.9/15 µM B16-F1, IC_50_: 3.9/16.1 µM WS1, IC_50_: 31/12 µM D-Ara(OH): B16-F1, IC_50_: 11/16.1 µM WS1, IC_50_: 47/12 µM - improvement in hydrosolubility	[132,133]
2.	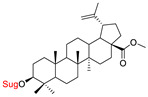	D-Glu L-Rha D-Ara	A549 DLD-1 B16-F1 WS1	resazurin reduction test (RTT), in vitro	D-Glc(OH): A549, IC_50_: 8.4/19 µM DLD-1, IC_50_: 3.9/25 µM B16-F1, IC_50_: 7.1/26 µM WS1, IC_50_: 9.3/19 µM	[133]
3.	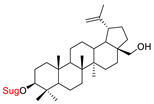	D-Glu, L-Rha D-Ara, D-Gal D-Man, D-Xyl	A549 DLD-1 WS1	resazurin reduction test (RTT), in vitro	improvement in hydrosolubility	[132]
4.	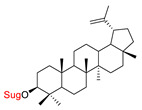	D-Glu L-Rha D-Ara	A549 DLD-1 B16-F1 WS1	resazurin reduction test (RTT), in vitro	L-Glc(OH): A549, IC_50_: 14/165 µM DLD-1, IC_50_: 14/125 µM B16-F1, IC_50_: 15/104 µM WS1, IC_50_: 13.3/63 µM D-Ara(OH): A549, IC_50_: 28/165 µM DLD-1, IC_50_: 50/125 µM B16-F1, IC_50_: 27/104 µM WS1, IC_50_: 15.8/63 µM	[133]
5.	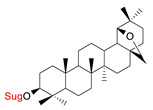	D-Glu L-Rha D-Ara D-Gal D-Man D-Xyl	A549 DLD-1 WS1	resazurin reduction test (RTT), in vitro	D-Glc(OH): A549, IC_50_: 31/>75 µM DLD-1, IC_50_: 40/>75 µM WS1, IC_50_: 40/>75 µM D-Gal(OH): A549, IC_50_: 30/>75 µM DLD-1, IC_50_: 40/>75 µM WS1, IC_50_: 30/>75 µM - improvement in hydrosolubility	[132]
6.	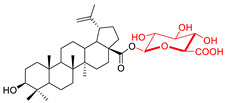		A549 DLD-1 WS1		- non-hemolytic, HD_50_ >100 µM - better hydrosolubility than BA - a good in vitro stability in phosphate buffer can be hydrolyzed in the presence of β-D-glucuronidase	[134]
7.	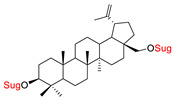	D-Glu L-Rha D-Ara	A549 DLD-1 MCF-7 PC-3 WS1	resazurin reduction test (RTT), in vitro	L-Rha(OH): A549, IC_50_: 1.9/3.8 µM DLD-1, IC_50_: 1.9/6.6 µM MCF-7, IC_50_: 1.7/23.3 µM PC-3, IC_50_: 1.8/17.9 µM WS1, IC_50_: 1.3/3.6 µM	[135]
8.	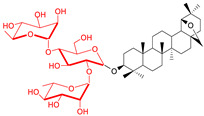	chacotriosyl	A549 DLD-1 MCF-7 PC-3 WS1	resazurin reduction test (RTT), in vitro	chacotriosyl: A549, IC_50_: 14/>50 µM DLA-1, IC_50_: 13/>50 µM MCF-7, IC_50_: 15/>50 µM PC-3, IC_50_: 13/>50 µM WS1, IC_50_: 9/>50 µM	[137]
9.	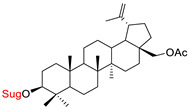	D-Man	CEM, MCF-7 A549, HeLa BJ-H-tert RPMI 8226 G 361	Calcein AM assay	D-Man(OH): CEM, IC_50_: 12.9/21.2 µM MCF-7, IC_50_: 35.5/>50 µM A549, IC_50_: 44.6/>50 µM HeLa, IC_50_: 42.8/>50 µM BJ-H-ter, IC_50_: 43.1/48.6 µM	[138]
10.	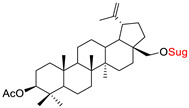	D-Man tri-D-Man	CEM MCF-7 A549 HeLa BJ-H-tert RPMI 8226 G 361	Calcein AM assay	D-Man(OH): MCF-7, IC_50_: 39.2/>50 µM A549, IC_50_: 44.6/>50 µM HeLa, IC_50_: 45.7/>50 µM BJ-H-tert, IC_50_: 35.6/48.6 µM	[138]
11.	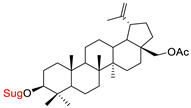	Glu			glucose-conjugated BN (B10) - apoptotic and non-apoptotic cell death coexist upon B10 treatment - it turns autophagy into a cell death mechanism	[141]
12.	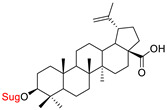	L-Rha di-L-Rha tri-L-Rha tetra-L-Rha	DLD-1 WS1	Hoechst test in vitro	L-Rha(OH): DLAD-1, IC_50_: 4.0/20 µM WS1, IC_50_: 33.0/36 µM di-L-Rha(OH): DLAD-1, IC_50_: 5.0/20 µM WS1, IC_50_: >100/36 µM tri-L-Rha(OH): DLAD-1, IC_50_: >100/20 µM WS1, IC_50_: >100/36 µM - in most cases, increasing the number of sugar units leads to reduction of cytotoxity	[142]
13.	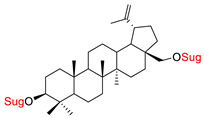	L-Arap l-Rhap L-Manp D-Idop	CEM MCF-7 HeLa G-361 BJ		cytotoxicity compared to BA 3-*O*- L-Arap-28-*O*-L-Arap: CEM, IC_50_: 2.6/40 µM MCF-7, IC_50_: 1.6/>50 µM HeLa, IC_50_: 1.2/47.6 µM G-361, IC_50_: 0.9/>50 µM BJ, IC_50_: 1.3/>50 µM 3-*O*- L-Rhap-28-*O*-L-Arap: CEM, IC_50_: 2.4/40 µM MCF-7, IC_50_: 1.7/>50 µM HeLa, IC_50_: 1.5/47.6 µM G-361, IC_50_: 1.1/>50 µM BJ, IC_50_: 1.5/>50 µM	[143]
14.	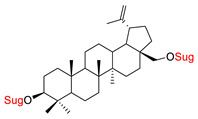		A549, NCI-H2087, NCI-H522, NCI-H1993 NCI-H1755, and LLC1		3,28-bis-*O*-L-Rham: IC50: 2.9 -5.9 μM - significantly inhibited tumor growth - can induce apoptotic cell death via disturbance of the mitochondrial electron transfer chain, reduced ROS production, and decreased membrane potential	[147]
15.	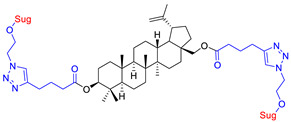	GalNAc	HepG2 Huh7 PC-3 A549	MTT-based cell viability assay	- high affinity for the asialoglycoprotein receptor (ASGPR) of hepatocytes (in silico) - moderate cytotoxicity and selectivity against HepG2 (IC_50_: 25.9 µM, for BN IC_5_0: 4.2 µM)	[150]

Ara: arabinose; Cel: cellulose; Fuc: fucose; Gal: galactose; Glu: glucose; Lac: lactose; Mal: maltose; Man: mannose; Xyl: xylose; Rha: rhamnose.

Another group of naturally occurring compounds is isoflavones, among which one of the best-studied compounds seems to be genistein. It is a natural phytoestrogen present in soybeans and native to *Southeast Asia* [151]. There are quite a few in vitro and in vivo studies described in the literature that have been carried out to better understand the mechanisms underlying the biomedical properties of genistein, especially its neoplastic potentials [152,153]. Genistein demonstrated many biomedical effects, such as antioxidant, antiproliferation, and anticancer activities [154]. The results of numerous in vivo and in vitro research demonstrated the pivotal genistein role as the molecules with high anticancer potential in varied types of cancer [155]. Genistein affects the arrest of the cell division cycle and apoptosis in multiple cancer cell lines both in in vitro and in vivo studies [156]. The anticancer effect of genistein may be related to its ability to inhibit enzymes such as protein tyrosine kinase and topoisomerase II, the possibility of inhibiting angiogenesis, numerous proapoptotic properties, as well as its effect on the estrogen receptor, which may be of particular importance in the hormone-responsive type of cancers [157]. The main limitations of genistein are its low water solubility, rapid metabolism in vivo, and, consequently, rapid excretion. Therefore, it seems advisable to introduce structural modifications that would improve its solubility, stability, and bioavailability [158]. An interesting subject seems to be the possible influence of this xenobiotic on the metabolic processes of normal physiological processes, as well as on pathologies such as tumorigenesis, through competition for receptor sites, signalling pathways, and active sites of enzymes [159]. Therefore, numerous known modifications of the genistein structure are aimed not only at improving its stability, solubility, and bioavailability but also at making it more selective than the parent compound. Sugars seem to be ideal candidates to modify the structure of genistein. Depending on the type of attached sugar moiety, the configuration of its anomeric center, as well as the presence or absence of groups that protect the sugar fragment, products with diversified lipophilicity and susceptibility to enzymatic degradation can be obtained. In particular, in natural sources, flavonoids, including genistein, are generally found in the form of glycosides [160].

**Figure 13:**
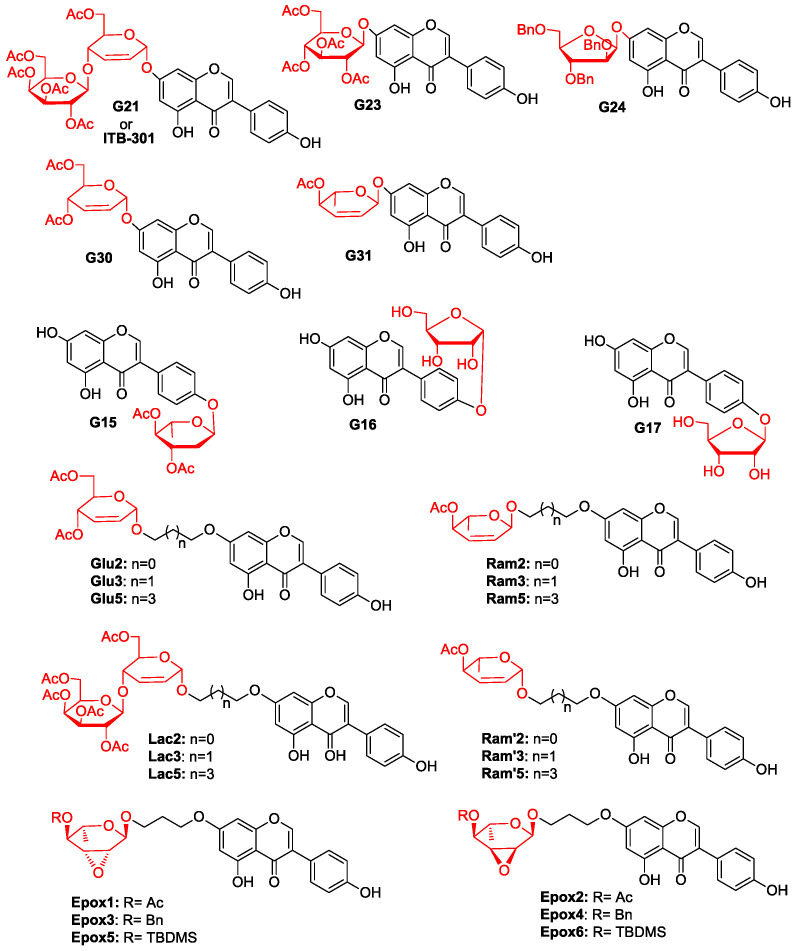
Genistein glycoconjugates.

Polkowski received several synthetic genistein glycosides (Figure 13, compounds G15, G16, G17, G21, G23, G24, G30, G31), which differ in the type of attached sugar, the presence or absence of protective groups in the sugar fragment, as well as the position of genistein glycosylation, and examined their antiproliferative and cytotoxic activity by comparing them with genistein and its naturally occurring glycoside genistin. Studies were carried out on four cell lines: HL-60, Colo-205, MCF-7, and PC-3. Among the glycoconjugates tested, those with higher lipophilicity (containing a protected sugar part) turned out to be more active, and among them, the most active turned out to be the G21 conjugate, which was definitely more active than genistein. The G30 derivative also showed good anticancer activity. Both glycoconjugates show some structural similarities: the presence of acetyl protective groups increasing lipophilicity, the same configuration of the glycosidic bond (α), the presence of a double bond between C2 and C3 carbons in the sugar, and the same substitution site in genistein (7-OH). The genistein glycosides containing unprotected sugar residues showed negligible activity, which can be attributed to their hydrophilic nature, making it difficult to penetrate the cell membranes. However, the lack of in vitro activity does not exclude its in vivo activity, where they may be degraded by enzymatic hydrolysis with the release of active aglycone [157]. The most active of the compounds tested, the G21 glycoconjugate, also known as ITB-301, was further intensively studied to determine the mechanism of action. DU 145 and HCT 116 cell lines were selected for this study. To assign the mechanism of antiproliferative activity of this compound, the influence of G21 on the cell cycle was determined. Additionally, it was microscopically determined the fraction of mitotic cells. Furthermore, it was checked if the mitotic block is related to changes in the mitotic spindle structure in immunofluorescent stained specimens. Research has shown that this compound acts as a microtubule destabilizing agent [161]. Studies of this compound have also been conducted in ovarian cancer cells (SKOv3). On the basis of the results, G21 was found to target microtubules, which depolymerize upon treatment with this genistein conjugate. The authors suggested that this compound could find application in the treatment of cancers characterized by MDR (multidrug resistance) [162]. Further research indicates that the mechanisms of cytotoxicity of genistein and its glycosidic derivative G21 are significantly different [163].

Conjugates of genistein and unsaturated pyranosides, in which the sugar was not linked to genistein by forming a glycosidic bond but through an alkyl chain of various lengths (Figure 13, compounds abbreviated Ram, Glu, and Lac) have also been described in the literature. The antiproliferative activity of these compounds was tested in vitro in HCT 116 cancer cell lines. Ram3 turned out to be the most active derivative that inhibited the cell cycle, interacted with mitotic spindles, and caused apoptotic cell death. For this derivative, the antiproliferative activity was assessed on a larger panel of tumor cells (11 lines including glioblastoma, breast, stomach, lung, prostate, and colon cancer) by comparing the IC_50_ values with the activity exhibited by genistein. These values determined for Ram3 were lower each time than for genistein, and for the MCF-7 and AGS lines, they were even lower [164]. The search for new epidermal growth factor receptor (EGFR) inhibitors for use in combination with radiotherapy in the treatment of solid tumors prompted scientists to investigate the antiproliferative potential of earlier described genistein derivatives G21, Ram2, Ram3, and Ram5 (Figure 13) used alone or in combination with ionizing radiation. Our research was carried out on the HCT 116 line. The results showed the tested compounds’ ability to decrease EGFR activation and suggests that these compounds are much more potent radiation sensitizers of cells to radiation than the parent isoflavonoid, genistein [165].

The positive results obtained for Ram3 became an inspiration to obtain a new line of genistein glycoconjugates containing 2,3-anhydrosugars linked to genistein through an alkyl chain (Figure 13, compounds abbreviated Epox). In vitro screening of antiproliferative activity of the new compounds was performed in HCT 116 cells. The most active were the derivatives Epox1, Epox4, and Epox5 (the determined IC_50_ values were 2.87 µM, 3.07 µM, and 4.57 µM, respectively). Each of them affected the cell cycle in a different manner. Epox1 blocked the cell cycle in the G2/M phase of a cycle, Epox4 prevented cells from entering S-phase, whereas Epox5 caused a significant increase of the frequency of sub-G1 phase, suggesting apoptosis [166].

**Figure 14:**
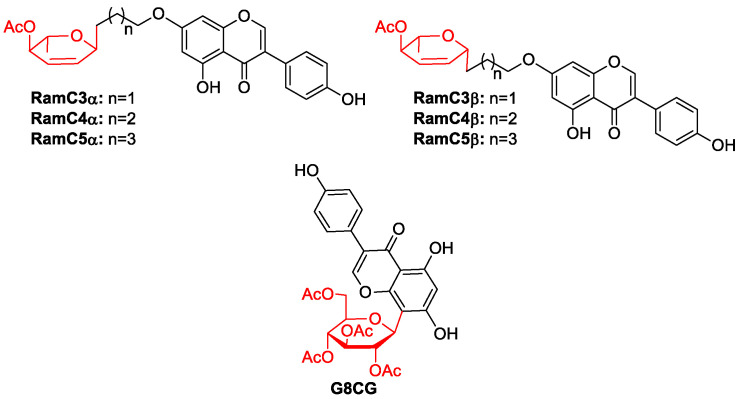
Genistein C-glycoconjugates.

One of the crucial problems of *O*-glycoside application in medicine and pharmation is related to the question of their stability in biological media. The *O*-glycosidic linkage is relatively labile in the presence of hydrolytic enzymes as well as in an acidic environment. Therefore, the replacement of the *O*-glycosidic bond with the *C*-glycosidic bond is often used to improve the stability of such connections. The situation is similar in the case of genistein glycoconjugates. Information can be found in the literature on genistein *C*-glycoconjugates that have been prepared and screened for anticancer activity. The structures of several of them are shown in Figure 14.

Two cancer cell lines, HCT 116 and DU 145, were used to determine the cytotoxicity of the six genistein derivatives shown in Figure 14, which differ in linker length and configuration in the anomeric center of the attached sugar unit (compounds abbreviated RamC). Four of the tested *C*-glycoconjugates of genistein (RamC3α, RamC4α, RamC5α, and RamC5β) demonstrated higher potency than the parent genistein. The new derivatives also significantly alter the cell cycle and cause mitotic perturbations not observed for genistein itself. The fact that up to three of them have the α-configuration at the sugar unit anomeric carbon is worth emphasizing. However, the length of the linker between the sugar fragment and genistein appears to have an equally significant impact on the demonstrated cytotoxicity [167]. On the basis of the research results described so far using an in vitro cell model, it can be noted that genistein glycoconjugates containing the *O*-glycosidic or *C*-glycosidic linkage show in most cases significantly higher antiproliferative activity in comparison to the genistein parent compound. Particularly noteworthy are derivatives that contain an attached 2,3-unsaturated sugar unit.

In the next work, studies on the structure–activity relationship between selected genistein derivatives (Figure 13, compounds abbreviated Ram and Ram’, Figure 14, compounds abbreviated RamC) and in vitro permeability by passive and active transport using biologically relevant membranes were described. Both in vitro and in vivo studies showed that genistein glycoconjugation allows for slower metabolism of genistein, and this was significantly influenced by the type of glycosidic bond. As expected, the *C*-glycosidic bond, because of its stability, allowed genistein’s metabolization to be significantly slowed down, which is crucial for its bioavailability and for extending its retention time in the body. The data presented can be summarized in such a way that genistein glycoconjugation can significantly modify the biological potency, bioavailability, and metabolic rate of the new drug [168]. An interesting fact is that naturally occuring genistein 8-C-glucoside (Figure 14, G8CG) isolated from *Lupinus luteus* L. (yellow lupine) is used for research on the possible application for the treatment and prevention of ovarian cancer. The effects of this compound alone or in combination with genistein on cultured human SKOv3 cancer cells were investigated. The results obtained showed that the combination of genistein and its *C*-glycoside G8CG inhibits the proliferation of the cells tested, induces apoptosis, evokes collapse of the mitochondrial membrane potential, and generates ROS. Genistein-G8CG combination can be indicated as a preparation that can potentially be used for ovarian cancer therapy [169].

Despite the beneficial effect of genistein glycoconjugation on its selectivity and pharmacokinetic parameters, it should also be mentioned that other compounds conjugated with genistein are able to improve its properties. An interesting example of such a successful conjugation is the covalent connection of genistein with a heptamethine cyanine dye IR 783. The resulting conjugate was tested in vitro for cytotoxicity both on a cancer cell line MCF-7 and healthy cells MCF-10A. The tested conjugate exhibited a lower IC_50_ value for MCF-7 cells in comparison to the parent genistein (10.4 μM versus 24.8 μM), and for healthy cells, the determined IC_50_ value was three times higher. In vivo test results indicated that such a conjugation definitely improved genistein pharmacological profile by cancer-cell-selective uptake and targeting [170].

In summary, the connection of bioactive compounds to appropriate modifying molecules (e.g., sugar units) has resulted in, in many cases, useful molecular hybrids with a high potential to treat cancer or compounds that are useful for the study of the mechanisms of action at the molecular level. In our opinion, the presented research is very important from the scientific point of view because they will help the researchers avoid future failures. Such a review could be a stimulus for other researchers to seek a biologically active compound with higher activity and optimum ADME-Tox properties with great potential to finally become clinically used therapeutics.

## 3. Sugar-Containing Drug Carriers

As mentioned above, a huge problem of modern oncology is the lack of sufficient selectivity of anticancer drugs, which is associated with their cytotoxicity in relation to diseased cells and healthy cells [6,45]. It comes to the poor solubility of drugs in the aqueous environment and the poor bioavailability [171,172]. There is also the problem of drug resistance associated with the elimination of drugs from cancer cells through appropriately specialized transporters [7,173]. The use of carriers that enable the delivery of the active substance in effective concentration to tumor cells without affecting normal cells may improve the anticancer effect of drugs. Specific delivery of drugs to tumor cells using nanoparticle carriers can occur by releasing the loaded substance from the carrier extracellularly into the tumor microenvironment or by intracellular drug release through endocytosis. The second variant occurs through the so-called active transport [174]. The connection of appropriate ligands to the carrier, which recognizes and binds to a specific receptor or antigen on the surface of cancer cells, makes it possible to increase the effectiveness of targeted drug administration. Binding of this ligand to a specific receptor allows it to enter the cell by the active transport [175]. There are many types of receptors known to be overexpressed in malignant cells. Many of them have been explored as docking sites for targeting anticancer drugs [176]. When selecting ligands for selective drug delivery in targeted therapy, the following receptors are most often taken into account: folate receptor [177], ASGPR [178], HER2 [179], CD44 [180], and GLUT transporters [181]. Ligands for many of these receptors are sugars, and hence carriers containing sugars in their structure are intensively studied for use in the selective delivery of anticancer drugs.

The number of publications focusing on drug carriers containing sugars, their synthesis, and applications in 2022 alone reached more than 1150 (search phrase: sugars for drug delivery, polysaccharide drug carriers, polysaccharides for drug delivery, according to the *Scopus database*).

### 3.1. Polysaccharide Drug Carriers

One of the drug carriers used is those obtained on the basis of polysaccharides, defined as polymers made of sugar units connected via a glycosidic bond. Polysaccharides, which are natural biopolymers, are one of the most common renewable raw materials, which is undoubtedly their advantage. This group of compounds includes both plant polysaccharides (cellulose, starch, pectins, gum arabic, alginate, agar, and carrageenan) and animal polysaccharides (chitin, hyaluronic acid, heparin, and glycogen), as well as those produced by microorganisms or fungi (agarose, dextran, and xanthan gum) [182]. They are commonly used in various industries, but the most interesting from the point of view of this work seems to be the use of these biopolymers to obtain drug carriers. Their undoubted advantages include the common occurrence and relatively simple acquisition from natural sources [183], biodegradability, low immunogenicity [184], the possibility of modifying functional groups depending on needs, the possibility of conjugating them with substances to be delivered to the target place [185], biocompatibility [186], and sensitivity to environmental stimuli, such as changes in pH, which allows them to be used as stimuli-responsive drug delivery systems [187]. In turn, the problem is the occurrence of differences between polysaccharides obtained from different batches of natural raw material and the appearance of the possibility of impurities that are difficult to remove. Interestingly, it was noticed that polysaccharides could be used as ‘adjuvants’ for cancer therapeutics. Some of them exerted antitumor activity through the cell cycle arrest, antiangiogenesis, apoptosis, and immunomodulation mechanisms. This may suggest that polysaccharides can not only be used as drug carriers but also could be utilized directly against cancer [188].

Obtaining polysaccharide nanocarriers loaded with an active compound can be performed in various ways. Cross-linking can be achieved, for example, by covalent cross-linking using glutaraldehyde and natural polycarboxylic acids, such as succinic acid, malic acid, tartaric acid, or citric acid, as well as different diamines [183]. Another method is ionic crosslinking using polyanions and polycations such as tripolyphosphate [189] and various bivalent cations, e.g., Ca^2+^, Ba^2+^, or Zn^2+^ [190,191]. Polysaccharide nanoparticles can also be formed by intermolecular–electrostatic interactions of oppositely charged polymers (polyelectrolyte complexation and complex coacervation). In this way, nanoparticles based on chitosan and carboxymethyl cellulose, dextran sulfate, or alginate are created [187]. Nanoparticles can also be formed by self-assembly of hydrophobically modified polysaccharides. Hydrophobic compounds, such as poly(ethylene glycol), long-chain fatty acids, poly(ε-caprolactone), or cholesterol, are used for modification [192]. The formation and loading of drug carriers can be carried out by the conjugation of the drug and polysaccharide, drug entrapment in hydrogels, or the formation of self-assembled polysaccharide drug-loaded nanoparticles. Examples of various junctions of drugs with polysaccharides are summarized in Table 3.

The connection of dextran and DOX can be indicated as examples of conjugate drug-polysaccharide. Dextran is an interesting biopolymer because of both its physicochemical characteristics and not very high cost. The hydroxy groups in dextran can be used to link to a drug via a covalent bond directly or a linker. For example, a pH-sensitive prodrug in which DOX was covalently decorated via a hydrazone bond on the dextran-based copolymer DEX-P (OEGMA-co-MGMA) prepared by one-step atom transfer radical polymerization (ATRP) using DEX-Br with OEGMA and MGMA monomer. The formed conjugate was able to self-assemble into a stable micelle and showed a high drug load capacity. Research on drug release showed a significant effect of pH on the amount of DOX released (72.43% DOX release at pH 5.0, 28.97% at pH 6.8, or only 15.71% at pH 7.4). Decreased drug release at neutral pH distinctive for normal tissues can allow for minimizing the side effects of the drug. In vitro studies have found that cell viability of HeLa and 4T1 cells significantly decreased when the drug concentration of DOXDT ranged from 0 to 10 μg/mL (Table 3, Entry 14). Furthermore, in vivo studies showed that the tumor volume of DOXDT-treated mice was smaller than in the control group, while systemic toxicity for normal tissues turned out to be minimal because of the pH-sensitive drug release [193]. Another work described that DOX was covalently conjugated via Schiff base linkages into the dextran-based nanogels, containing disulfide bonds formed in the reaction between dextran polyaldehyde and cystamine in inverse water-in-oil microemulsion. In this way, dual-stimuli responsive polymeric nanoparticles that can respond to acidic and reductive (GSH) environment sensitive were obtained. The DOX release profiles of the obtained nanogel were monitored at different pH values and GSH contents. The most rapid release of DOX was detected as dual stimuli of pH 5.0 and glutathione at a concentration of 10 mM were simultaneously applied. In these conditions, more than 88% of DOX was released in 158 h. Cell viability assayed for the human non-small cell lung carcinoma cell line (H1299) and cervical cancer cell line (Hela) after 48 h of incubation with DOX and DOX-loaded nanogel of 4 μg/mL concentrations was 14% and 12%, respectively. In turn, cells treated with nanogels without DOX for 48 h did not show significant cytotoxicity up to 320 μg/mL concentration. Observed in in vitro studies, the antitumor effects of DOX-loaded nanogels to the tested tumor cell lines may result from the effective DOX release induced by the intracellular low pH and high GSH level. The results obtained prove that tested drug-loaded nanogels tested could be applied as microenvironment-responsive drug delivery system for cancer therapy [194].

An interesting example of the use of hydrogel polysaccharide carriers is a nanocomplex constructed from alginate hydrogel coloaded with cisplatin and gold nanoparticles (AuNPs) for simultaneous drug delivery and computed tomography imaging. The therapeutic potency of the obtained nanocomplex was tested in vitro using CT26 cells derived from mouse colon adenocarcinoma. Cytotoxicity was determined using the MTT assay. Cells were also treated with the above-mentioned nanocomplex to image in a computed tomography scanner, and the contrast enhancement was assessed due to the presence of nanocomplex. The cytotoxicity results showed a higher therapeutic effectiveness of the nanocomplex compared to that of the free cisplatin. In addition, the studied nanocomplex increased the brightness of computed tomography images in comparison with that obtained with the use of uncoated AuNPs. This shows that alginate coating can facilitate the cell membrane crossing of the nanocarriers, resulting in enhanced drug delivery to tumor cells [195].

An example of a bioactive polysaccharide that is used as a carrier of anticancer drugs is cell surface glycoprotein CD44 binding hyaluronic acid. CD44 is a transmembrane glycoprotein, also known as P-glycoprotein 1, which has been found to be overexpressed on the surface of cancer cells in breast, ovarian, lung, and stomach cancers [196]. An important observation is that the expression of this glycoprotein in healthy cells is significantly lower. Therefore, overexpressed in tumor cells, the CD44 receptor can be targeted by drug-loaded nanoparticles coated with hyaluronic acid, and hyaluronic acid appears to be the perfect carrier to achieve selective drug delivery. An example of this type of targeted therapy is the use of hyaluronic acid-based nanocarriers cross-linked with cisplatin and loaded with DOX. Its efficiency was tested against CD44^+^ breast cancer cells (4T1) in both the in vitro and in vivo systems, as well as in CD44 normal fibroblast cells (NIH-3T3). The first stage of the research was to determine the rate of drug release at different pH values corresponding to both the tumor microenvironment and physiological conditions. After 72 h, drug release from cross-linked micelles was less than 35% at pH 7.4. However, at pH 6.8 or 5.5, drug release increased to approximately 50% and 80%, respectively. In further studies, both cell lines were treated with free drugs and drug-loaded micelles. Time- and dose-dependent cytotoxicity was observed in both cases. Drug-loaded micelles showed stronger cellular growth inhibition than free drugs against 4T1 (CD44^+^) breast cancer cells, while no significant differences in growth inhibition were observed between drug-loaded micelles and free drugs in control cells characterized by the lack of expression of CD44 receptors. In in vivo studies, micelles exhibited stronger inhibitory effects and lower systemic toxicity than free drugs in a mouse model with mammary cancer 4T1. The results obtained confirm the advantages resulting from the use of a carrier targeted at CD44 receptors, hyaluronic acid, for the delivery of drugs in the treatment of breast cancer [197].

Although in the current literature, one can find a huge amount of information on the preparation and application of polysaccharide-based drug nanocarriers, these few discussed examples of the use of polysaccharide carriers for the selective delivery of anticancer drugs are enough to confirm the effectiveness of the adopted strategy aimed at reducing the systemic toxicity of commonly used anti-cancer drugs and the nuisance of side effects accompanying therapy with free drugs. Other selected examples of successful application of polysaccharide-based targeted drug delivery systems are summarized in Table 3.

**Table 3:** Polysaccharide-based carriers for anticancer drugs.

Entry	Polysaccharide	Type of Drug Binding	Anticancer Drug	Type of Anticancer Activity Studies	Activity/Properties	Ref.
1.	Chitosan (low molecular weight chitosan, LMWC)	conjugation via succinic anhydride	PTX	B16F10 female C57BL6 mice, melanoma cells; in vivo	IC_50_ values comparable to parent PTX	[198]
2.	Chitosan/10% dextran sulfates	encapsulation	DOX	A375 and C26; in vitro	the presence of dextran sulfate allowed the DOX-loaded carrier to maintain cytotoxicity at a level comparable to free drug	[199]
3.	*N*,*O*-carboxymethyl chitosan (*N*,*O*-CMCS)−guar gum (*N*,*O*-CMCS/MAGG)	pH-responsive swelling of hydrogels	DOX	MCF-7, in vitro	67% DOX release after 5 days in pH of 5.5 32% DOX release at pH of 7.4 IC_50_: 98.45 μg/mL	[200]
4.	Chitosan nanoparticles (CCNP)	encapsulation in nanoparticles using an ionic gelation	CDDP	MCF-7, in vitro	43.80% CDDP release in 6 h IC_50_: 4.085 μg/mL	[201]
5.	Chitosan nanoparticle surface linked to rituximab (mAbCCNP)	encapsulation in nanoparticles using an ionic gelation	CDDP	MCF-7, in vitro	22.52% CDDP release in 4 h no cytotoxicity	[201]
6.	Chitosan	encapsulation in nanoparticles using an ionic gelation	5-FU	SGC-7901, in vitro pharmacokinetic studies; in vivo	76% release in the first 0.7 h, sustained release 0.7 to 8.0 h the same inhibitory effect as 5-FU injection half-life increased after intravenous administration compared with 5-FU solution, in vitro	[202]
7.	Chitsan (CS-NPs)	encapsulation in nanoparticles using an ionic gelation	GEM	OVCAR-8, in vitro	77.27% drug release in 24 h cytotoxicity nanoparticles loaded with drug comparable to parent drug	[203]
8.	Chitosan chemical conjugated with epidermal growth factor receptor variation III (CS-NPs-EGFRv)	encapsulation in nanoparticles using an ionic gelation	GEM	OVCAR-8, in vitro	the cytotoxicity of CS-NPs-EGFRv loaded with the drug is higher than parent drug	[203]
9.	Chitosan (CHT)	conjugation via succinic anhydride (SA), nanoparticles prepared by the precipitation dialysis method	DTX	MDA-MB-231, in vitro	the release of the drug was pH dependent, higher in pH = 5.6 than in pH = 7.4 IC50 of DTX-SA-CHT: 4.68 μg/mL IC50 of DTX: 37.50 μg/mL pharmacokinetic studies show that bioavailability increases with increased half-life and decreased elimination of drug from the biological system	[204]
10.	Pullulan/Chitosan 1:2 (NEPl2-Cs 1:2)	nano-emulsion	DOX	A375 BRAF and HaCaT; in vitro	increased induction of melanoma cell apoptosis and a definite increase in cytotoxicity against A375 cells in case of drug-loaded nano-emulsion application in comparison to free DOX	[205]
11.	Alginate/Chitosan	encapsulation in nanoparticles using two-phase system	DOX	4T1, in vitro	at pH 5.5, 70% of DOX has been released within 8 h time point, 90% of the drug was released within 24 h IC_50_ of nanoparticles with DOX: 0.15 μg/mL IC_50_ of DOX: 0.13 μg/mL	[206]
12.	Alginate (ALG)	PTX -loaded nanoparticles prepared by the nano-emulsification polymer cross-linking method	PTX	Cell cycle analysis, breast cancer cells, in vitro	PTX -loaded nanoparticles inhibit cellular proliferation, block cell cycle progression, and induce apoptosis in cancer cells the percentage of apoptotic cells in untreated cells increased from 11% to 83% after treatment with PTX nanoparticles	[207]
13.	Alginate (ALG)	co-loaded hydrogel (ACA)	CDDP and AuNPS	CT26, in vitro	the ACA nanocomplex is more effective than CDDP: the ACA nanocomplex at a concentration of 5 µg/mL (per cisplatin) and 20 µg/mL of free cisplatin resulted in the same cytotoxicity (survival rate: 66%) the ACA nanocomplex increased the brightness of computed tomography images and contrast to noise ratio	[195]
14.	Dextran as a copolymer component DEX-P(OEGMA-co-MGMA)	DOX covalently decorated on the copolymer nanocarrier by conjugation via *a* pH-responsive hydrazone bond	DOX as conjugate (DOXDT)	4T1, HeLa human cervical cancer cell line, in vitro Balb/C mice bearing 4T1 tumor, in vivo	pH-dependent drug release (higher in an acidic environment) cell viability of HeLa and 4T1 cells significantly decreased in the presence of DOXDT, in vitro the tumor volume of DOXDT treated mice was smaller than in control group (control group: increasing from 139.74 to 1376.35 mm^3^ after 14 days; DOXDT group: increasing to 296.63 mm^3^)	[193]
15.	Dextran (DEX-SS)	dextran-based nanogels (DEX-SS) created by Schiff base formation between polyaldehyde dextran (DEX-CHO) and cystamine DOX conjugated into DEX-SS nanogels via Schiff base linkages	DOX as conjugate	H1299 and Hela, in vitro	DOX-loaded dextran nanogels penetrate cancer cells and, under the influence of both the environmental pH and the amount of GSH, release the drug	[194]
16.	Dextran (DEX)	negatively charged dextran-based dual conjugates with different length linkers	DTX and DHA as conjugate	HTB-177, MCF-7, and 4T1 mouse breast cancer cells, in vitro 4T1 breast cancer cells in BALB/C mice, in vivo	in vitro: comparable activity of DTX and its conjugate (DEX-DHA-DTX) the conjugates improved drug solubility and increased the amount of drug within tumor cells, while its concentration in healthy cells was lower than that with free DTX in vivo: the conjugate caused tumor disappearance in mice, no side effects	[208]
17.	Dextran oxidised to dicarboxydextran (DXA)	CDDP-crosslinked DXA nanogels	CDDP	A2780, A2780/CP CDDP-resistant subline, A549, 22Rv1, PC-3, in vitro	CDDP conjugates with high-Mw DXA showed up to four times increased anticancer efficacy against malignant prostatic cell lines than free CDDP, and significantly inhibited ovarian cancer cell migration	[209]
18.	Hyaluronic acid (HA)	dual drug-loaded HA micelles (HA-DOX-CDDP)	DOX and CDDP	4T1, NIH-3T3, in vitro 4T1-xenografted Balb/c mice, in vivo	HA-DOX-CDDP micelles exhibited in vitro: increased drug release at acidic pH, better drug uptake and increased antiproliferative activity than in case of free DOX in vivo: less systemic toxicity and greater efficacy than free DOX	[197]
19.	Hyaluronic acid conjugated with casein (HA/casein 3:1)	hyaluronic acid -coated paclitaxel-loaded casein nanoparticles (HA-PTX-Cas NPs)	PTX	A375, in vitro male hairless mice HRS/J, in vivo	compared to uncoated PTX-Cas NPs, HA-PTX-Cas NPs reached a higher entrapment efficiency (93.1%) and exhibited satisfactory stability, HA-PTX-Cas exhibited a high efficiency (61.3%) in inhibiting A375 tumor mice experiments showed 74.6% tumor inhibition of HA-PTX-Cas by intravenously administration	[210]
20.	Hyaluronic acid (HA)	HA conjugates of DOX and GEM with different linkers	DOX and GEM	MDA-MB-231, 4T1, in vitro BALB/c mice bearing 4T1 tumor, in vivo	polymer conjugates released GEM faster than DOX more effective in killing triple negative breast cancer cells in vitro, more effectively inhibited the growth of the 4T1 tumor model in vivo than free DOX and GEM after intravenous and subcutaneous injection	[211]
21.	Hyaluronic acid coated B-mR9	nanoparticles coated with HA branched modified nona-arginine (B-mR9), composed of redox-cleavable disulfide bonds and complexed with MTX (B-mR9-MTX/HA)	MTX	NCI-H460, MCF-7, NIH-3T3, in vitro female, 6 weeks old BALB/c nude mice, in vivo	B-mR9-MTX/HA in vitro: improve drug delivery to cancer cells in vivo: better biodistribution, long retention in the body, and high tumor inhibition ability	[212]
22.	amine-functionalized nanocrystalline cellulose grafted folic acid/magnetic nanoparticles (AF-NCC/Fe_3_O_4_ NPs)	encapsulating DOX in AF-NCC/Fe_3_O_4_ NPs	DOX	Saos-2, in vitro	high encapsulation efficacy high stability at physiological pH high rate of drug release at low pH increased therapeutic effects compared to free DOX	[213]
23.	Thiolated heparin	polyion complex crosslinking by oxidation under atmosphere	DOX	MDA-MB-231 and HUVEC, in vitro 4 weeks old female Balb/c nude mice, in vivo	pH and GSH dual-sensitive drug release behavior in vitro polyion complex showed improved, compared to free drugs, anti-tumor performance and lower side effect to normal tissue both in vitro and in vivo	[214]

5-FU: 5-fluorouracil, AuNPs: gold nanoparticles, CDDP: cisplatin, DHA: docosahexaenoic acid, DOX: doxorubicin, DTX: docetaxel, GEM: gemcitabine, MGMA: methyl glycolate methacrylate, MTX: methotrexate, NPs: nanoparticles, OEGMA: methyl ether methacrylate, PTX: paclitaxel.

### 3.2. Glycopolymers

Glycopolymers are synthetic polymers with attached sugar units. Information on their synthesis was published in the 1970s [215], but their exceptionally dynamic development has been observed only since the 1990s. This development was largely the result of a desire to combine nanotechnology with carbohydrate chemistry to achieve synergy in the advantages of each of these groups of compounds. Created as a result of this type of ‘combination’, various types of glycopolymers have a wide range of applications, particularly medical applications, especially for drug delivery and release systems. These possible application glycopolymers are based on the ability to mimic the biological functions of natural oligosaccharides and glycans in lectin recognition processes [216]. Lectins are glycoproteins that possess the ability to specific sugar moieties [217]. Many epithelial tumors, such as colon, thyroid, and breast carcinomas, express galectin-1 and galectin-3. Galectins share an affinity for β-galactoside moieties. Another type of lectins are selectins, a group of cell-adhesion molecules, which include L-selectin, E-selectin, and P-selectin. This group binds to carbohydrate ligands in a calcium-dependent manner and play critical roles in host defense and in tumor metastasis by their ability to mediate cell–cell interactions. P-selectin binds to sulfated proteoglycan and heparin [218].

The interaction between lectins and sugar is rather weak because it is based on hydrogen bonding, van der Waals’ interactions, and hydrophobic stacking at the molecular level, but it can be strengthened by the simultaneous effect of several carbohydrates located close to each other [219]. This fact became the driving force for the design of glycopolymers containing more sugar units. Glycopolymers can be prepared in two ways. In the former, a monomer contains an attached sugar moiety that can be polymerized using various techniques. In another possible variant, a previously prepared polymer containing the appropriate reactive group can be functionalized with sugars [220]. When designing glycopolymers to be used as targeted carriers of anticancer drugs, the specific features of cancer cells and the tumor microenvironment should be taken into account (such as temperature, pH, and glutathione concentration). This will maintain the stability of the carriers under physiological conditions and will allow the release of the drug under the influence of factors typical for cancer. In this way, the so-called stimuli-responsive polymers are designed. In turn, the attached sugar fragments are designed to improve the solubility of the carrier and its targeting of cancer cells [221].

The way in which the drug is bound by the glycopolymer carriers can be different. The drug can be bound to a polymer or incorporated sugar by a covalent bond but also can be encapsulated in a nanocarrier. Drugs can be released by different agents and the mechanism of their selective delivery and release can be different depending on the glycopolymers used.

#### 3.2.1. Glycopolymers with Encapsulated Drug

In the literature, most of glycopolymers for drug delivery are carriers with drugs trapped in micelles; examples of such glycopolymers presented in this article are in Table 4. Entries 1, 3, 5–10, 15, 17–20, 22, 23, 25–29, 31, 33, 35, 38.

##### pH-Responsive Glycopolymers

One possible drug release mechanism is based on the carrier sensitivity to changes in pH, which allows the use of physiological differences between malignant and normal cells or between tumors and normal tissues. The drug delivery process is based on the difference between, considered neutral, the physiological pH = 7.4 and the acidic pH of the cancer microenvironment. The pH of the tumor microenvironment is reported to be in the range of 6.2 to 6.9 and even sometimes lower [221]. Nanocarriers designed to respond to pH changes usually contain acids-labile groups incorporated into a polymer structure such as acetal/ketal, hydrazone, orthoester, etc. [222,223,224,225]. These types of carriers remain stable until they reach the tumor and can respond to the acidic microenvironment of cancer cells by disassembling and changing size, shape, or carrier surface charge [226,227,228,229,230], which ultimately leads to the release of the encapsulated drugs in a controlled manner. Nanocarriers designed to be responsive to specific pH values can target a neoplastic area in the body to release their encapsulated anticancer agent, maximizing therapeutic impact and minimizing side effects. Numerous examples of glycopolymer nanocarriers can be found in the literature based on the described criterion of selective release of the active substance, and several examples are presented in Table 4, Entries 2, 3, 5–7, 13, 19, 31, 34. [231,232,233,234,235,236,237,238,239]. One example is the nanocarrier PEG-b-PGAMA-b-PDEA (Table 4, Entry 6) consisting of poly-(2-(diethylamino)ethyl methacrylate) (PDEA), as a pH-responsive cationic polymer with high biocompatibility, poly(D-gluconamidoethyl methacrylate) (PGAMA) and poly(ethylene glycol) (PEG), the most commonly used hydrophilic segment used in drug delivery systems. PEG-b-PGAMA-b-PDEA is a glycopolymer carrier with the hydrophobic drug DOX contained in the micellar core. This nanocarrier showed self-assembly under normal conditions at pH = 7.4 and at a slightly acidic pH in the range of 6.5–5.0, doxorubicin was beginning to be released. The effect of micelle structure on drug loading and release behavior was studied, which indicated a promising application for controlled drug release [234]. Another pH-responsive glycopolymer is poly(3-O-methacryloyl-D-glucopyranose)-b–poly(2-(4-formylbenzoyloxy)ethylmethacrylate) PMAG-b-PFBEMA-TBO-DOX (Table 4, Entry 3). This carrier with toluidine blue (TBO) and DOX is anchored on the surface of gold nanoparticles (AuNPs) and has a double effect because it has reactive oxygen species (ROS) generating ability under 630 nm light and release of DOX under the acidic pH of tumor cells. An in vitro cytotoxicity study in the MDA MB 231 cell line with this carrier gave good results and showed potential for the use of this drug using chemo- and photodynamic therapies [232]. Poly(2-deoxy-2-methacrylamido-D-glucose-co-2-hydroxyethyl methacrylate)-b-poly(β-amino ester) [P(MAG-co-HEMA)-b-PBAE] (Table 4, Entry 7) also exhibited active cancer cell targeting potential and pH response. P(MAG-co-HEMA) block is hydrophilic and, because of glucose residues, is a cancer cell targeting block, while PBAE was used as a pH-sensitive hydrophobic and degradable segment. The drug delivery potential was evaluated using cell viability assays for the non-cancer HUVEC cell line and the U87-MG glioblastoma cell line. The glycopolymer was not toxic in non-tumor HUVEC cells, being toxic only to the U87-MG cell line. This presents potential for cell-targeted cancer treatment [235].

##### Light-Responsive Glycopolymers

Light-responsive polymeric carriers have the ability to release the drug influenced by light, and it can be performed in the absence of additional chemical substances that initiate the reaction when in their structure, they contain compounds capable of isomerization induced by UV radiation, for example, azobenzene derivatives [240]. An example of this type of material suitable for the targeted delivery of anticancer drugs is a fluorescent glycopolymer-based nanogel PAG-*b*-PFMA poly(2-(acrylamido)glucopyranose)-*block*-poly(furfuryl methacrylate) (Table 4, Entry 1). In order to impart fluorescent activity, GQD gelatin quantum dots were introduced into the nanogel and the anticancer drug DOX, which was introduced inside the micellar system. By monitoring the fluorescently active glycopolymer nanogel, it was shown that it has potential as a drug carrier with a targeted anticancer effect due to the fact that it effectively delivers the drug to breast cancer cells (MBA-MD-231) and is a noncytotoxic substance towards healthy cells. The DOX-loaded nanogels PAG_20_-*b*-PFMA_32_ had an IC_50_ value of 0.908 μM, whereas free DOX showed an IC_50_ at the level of 0.631 μM. Human dermal fibroblast cells were also tested to see if the cytotoxic effect occurs in relation to healthy cells. Only at higher concentrations a cytotoxic effect was found, indirectly indicating the target specificity of the glycopolymer nanogel [241]. Another photosensitive glycopolymer (Table 4, Entry 27) was prepared using azobenzene methacrylate (AzoMA) and 2-(2,3,4,6-tetra-*O*-acetyl-β-D-galactopyranosyl)ethylmethacrylate (*b*-AcGalEtMA). These are galactose-based light-responsive block copolymers, which were self-assembled into micelles and were used for the delivery of Nile red to melanoma cells. The loading of the hydrophobic dye Nile red and the efficient cellular uptake by human melanoma cells (A375) were demonstrated. It is suspected that the specific interaction of galactose with galectin 3 receptors overexpressed on melanoma cells promotes uptake. In cell studies, the unloaded micelles showed low cytotoxicity and the Nile red-loaded micelles showed high cellular uptake in human melanoma cells, demonstrating their usefulness as a potential drug delivery system for this type of cancer [240]. Another example of this type of carrier is P(BOB-HA)-P(Fru)-PDS/Vc hydrogel (Table 4, Entry 16), which consisted of benzoxaborole (BOB) modified hyaluronic acid (BOB-HA) and fructose-based glycopolymer (PolyFru), with photosensitiser perylene diimide zwitterionic polymer (PDS) and ascorbic acid (Vc), a dynamically reacting light covalent hydrogel operating in the near infrared (NIR), which reaches the tumor site. The hydrogel disintegrates under the influence of irradiation with light with a wavelength of 660 nm as a result of breaking dynamic BOB-sugar covalent bonds. The study results showed that after irradiation with light, the hydrogel was degraded, allowing doxorubicin bound to it to be released [242].

##### Thermoresponsive Glycopolymers

The application of thermoresponsive polymers is one of the main approaches to the preparation of stimuli-responsive polymers. Changes in their physical properties observed in response to changes in temperature make them candidates for drug delivery in cancer treatment because, in the human body, the temperature of cancer cells is different from that of healthy cells [221]. The example of glycopolymer of this type is poly(2-lactobionamidoethyl methacrylamide)-b-poly(sulfobetaine methacrylate) PLAMA-*b*-PSBMA-*b*-PNIPAM (Table 4, Entry 39). These types of lactose-decorated dual responsive star-shaped nanogels are hepatoma targeted and could be used as hepatoma-specific anticancer drug delivery vehicles for cancer chemotherapy. The IC_50_ value of DOX-loaded nanogels was significantly lower in human hepatoma cells (HepG2) compared to nonhepatic HeLa cells. These results showed that sugar containing (PLAMA-*b*-PSBMA)-*b*-PNIPAM nanogels enhanced and selected DOX delivery to HepG2 cells as a result of the specific binding of the lactose residue to ASGP-R so that multi-reactive (thermo- and redox sensitive) lactose-functionalized nanogels could potentially be used for the delivery drugs against liver cancer [243]. Poly(*N*-isopropylacrylamide-*co*-6-*O*-vinyladipoyl-D-glucose)-*b*-poly(*N*-isopropylacrylamide) P(NIPAM-*co*-OVAG)-*b*-PNIPAM) (Table 4, Entry 4) is another example of thermosensitive polymer. The block glycopolymer was characterized by good cellular biocompatibility and minimal cytotoxicity. Glycopolymer micelles with Con A could be used to induce apoptosis in SMMC-7721 human hepatoma cells, due to their temperature-sensitive properties and protein recognition. They have potential applications in cancer cell targeting, as drug release carriers, and in clinical diagnosis [244]. Thermoreactive glycopolymer, poly(diethylene glycol methacrylate)-*block*-poly(6-*O*-vinyladipoyl-α-D-galactose) (PDEGMA-*b*-POVNGA) and modified gold nanoparticles AuNRs with Au-S linkages were used to create a novel focus on hepatoma therapeutic glycAuNR (Table 4, Entry 28). In vitro studies have shown that the synthesized carrier can significantly improve the cytocompatibility of AuNR and target hepatocellular carcinoma cells, thanks to galactose that recognizes the overexpressed asialoglycoprotein receptor in hepatocellular carcinoma cells. The photothermal test proved that the modified nanocarrier can conduct highly effective photothermal treatment of cancer cells in vitro after laser irradiation [245,246].

#### 3.2.2. Glycopolymers with Bounded Drug

Examples of glycopolymers in which the drug is bound to the embedded sugar are three block glycopolymers poly(ethylene glycol)-*block*-poly(gluconamido ethyl methacrylate) PEG_113_-*b*-PGAMA_20_, poly(ethylene glycol)-*block*-poly(styrene)-*block*-poly(gluconamido ethyl methacrylate) PEG_113_-*b*-PS_50_-*b*-PGAMA_20_, and poly(ethylene glycol)-block-poly(2-(diethyl amino) ethyl methacrylate)-*block*-poly(gluconamido ethyl methacrylate) PEG_113_-*b*-PDEA_50_-*b*-PGAMA_20_ (Table 4, Entry 5). These glycopolymers have the ability to charge the anticancer drug BTZ to a physiological pH of 7.4 using the conjugation method by the dynamical covalent complexation between glucose and boronic acid, which resulted in the attachment of BTZ to the micelle shell and physical encapsulation by accumulation in the core of micelles as a result of the hydrophobicity of drugs. These glycopolymers, with the ability to self-assemble in micelles, provide a promising polymer nanocarrier system for bortezomib, with extended drug release and accurate controlled release in vivo [233]_._

Poly(methyl methacrylate-*b*-((2-methacryloxyethoxy) benzaldehyde-*b*-fructomethacrylate)- zinc(II)phthalocyanine P(MMA-*b*-MAEBA-*b*-FrucMA)-ZnPc is a glycopolymer conjugated to the compound ZnPc-N_3_ and also covalently conjugated with DOX in the side groups of the P(MAEBA) via the pH sensitive imine bond (abbreviated as GNPs-ZnPc/Dox). In vitro studies were performed to determine the cellular uptake and anticancer efficacy of GNPs-ZnPc/Dox. Compared to free DOX, human breast cancer cells treated with glycopolymer showed higher antitumor activity by targeting GLUT5 (Table 4, Entry 13) [236].

P(ManMac)-*r*-(MAA), where ManMac is a mannose methacrylate glyconomer, and MAA is a methacrylic acid, is also described in the literature as an example of a glycopolymer with a bonded drug. Thiol-terminated glycopolymers were grafted onto AuNP gold nanoparticles. The DOX was linked to the glycopolymers and thus obtained through a pH-sensitive hydrazone bond in the presence of cysteine and a crosslinking agent. Gold nanoparticles coated with glycopolymer have been designed as pH-responsive anticancer drug carriers that release the drug under acidic conditions. This carrier has a therapeutic effect and higher toxicity in human neuroblastoma cells compared to healthy cell lines. The DOX molecule combined with Man-tagged AuNP/polymer molecules with a pH-sensitive hydrazone bond was evaluated for its in vitro release profiles and to investigate the potential use of these molecules as drug delivery systems (Table 4, Entry 31) [238].

A hybrid drug delivery system of glycopolymer and nanodiamonds presented in Table 4, Entry 14, was prepared by grafting amonafide-conjugated glycopolymers on the surface of nanodiamonds with oxime- P(MAFru)-*b*-P(3-VBA)-*co*-MMA-AMF poly(1-*O*-methacryloyl-2,3:4,5-di-*O*-isopropylidene-β-D-fructopyranose)-*b*-poly(3-vinylbenzaldehyde-*co*-methyl methacrylate). The anticancer drug is conjugated to the polymer by an imine bond, and the glycopolymer with the drug is grafted onto the surface of the aminooxyfunctionalized nanodiamonds. The prepared drug delivery system can effectively deliver amonafide to breast cancer cells and significantly inhibit the viability of these cells [247].

**Table 4:** Glycopolymer-based carriers for anticancer drugs.

Entry	Polymer	Attached Sugar	Linker (Binding Type)	Drug	Methodology	Results (IC_50_ or Percent Inhibition)	The Postulated Mechanism	Ref.
1.	PAG-*b*-PFMA	Glu	ester bond	DOX	MBA-MD-231 MTT assay, in vitro	Free DOX MBA-MD-231 IC_50_: 0.631 μM Glycopolymer MBA-MD-231 IC_50_: 0.908 μM	REDOX-responsive glycopolymer	[241]
2.	PMAG-*b*-P(Lys-*co*-Phe)	Glu	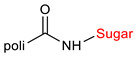	PTX	MCF-7, A549 CTB assay, in vitro	PTX-LANS (commercially available formulation with PTX) MCF-7 IC_50_: 4 ± 1 ng/mL A549 IC_50_: 2.0 ± 0.3 ng/mL PMAG-*b*-P(Lys-*co*-Phe) MCF-7 IC_50_: 4.1 ± 0.5 ng/mL A549 IC_50_: 4.4 ± 0.6 ng/mL	pH sensitive	[231]
3.	PMAG’-*b*-PFBEMA	Glu	ester bond	DOX AuNPs	MDA-MB-231 MTT assay, in vitro	-	GLUT transporters, pH sensitive	[232]
4.	P(NIPAM-*co*-OVAG)-*b*-PNIPAM	Glu	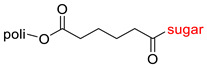	ConA	SMMC-7721 MTT assay, in vitro	-	ConA receptor thermosensitive glycopolymer	[244]
5.	PEG-*b*-DEA-*b*- GAMA PEG-*b*-PGAMA PEG-b-PS-*b*-PGAMA	Glu	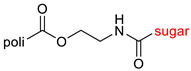	BTZ	L929	-	pH-responsive glycopolymer	[233]
6.	PEG-*b*-PGAMA-*b*-PDEA	Glu	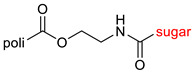	DOX	in vitro	-	pH-sensitive micelles	[234]
7.	P(MAG-co-HEMA)-b-PBAE	Glu	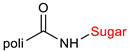	DOX	U87-MG, MTT assay in vitro	-	pH-responsive glycopolymer	[235]
8.	GP-Gluc-CDDP	Glu	ester bond	CDDP	OSC-19, U87MG, in vitro cytotoxicity assay	-	GLUT transporters	[248]
9.	p(1-O-MAFru)-*b*-PMMA	Fruc	ester bond	curcumin	MCF-7, RAW 264.7, SRB assay, in vitro	glycopolymer MCF-7 IC_50_: 15.2 µM RAW 264.7 IC_50_: 5.7 µM	GLUT transporters	[249]
10.	P(1-O-MAFru)-*b*-PMMA	Fruc	ester bond	PTX	MDA-MB-231, MCF-7, flow cytometry, in vitro	glycopolymer MDA-MB-231 IC_50_: 4.48 ± 0.10 µM MCF-7 IC_50_: 27.57 ± 0.50 µM	GLUT transporters	[250]
11.	P(FrucMA-*b*-MAEBA)-Py	Fruc	ester bond- sugar 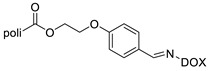	DOX (conjugate)	MCF-7, MDA-MB-231 MTT assay, in vitro	Apoptotic effect (%) for 24 h of glycopolymer: MCF-7: 85.00% MDA-MB-231: 81.24% glycopolymer with folic acid MCF-7: 87.46% MDA-MB-231: 96.58% free DOX: MCF-7: 42.68% MDA-MB-231: 72.80%	GLUT transporters	[251]
12.	P(FrucMA-*b*-MAc)-GEM	Fruc	ester bond	CDDP, GEM	MDA-MB-231, CCD-1079Sk, in vitro	glycopolymer CCD-1079Sk IC_50_: 125.68 ± 0.011 μg/mL MDA-MB-231 IC_50_: 31.51 ± 0.021 μg/mL	pH-sensitive glycopolymer, GLUT transporters	[252]
13.	P(MMA-*b*-MAEBA-*b*-FrucMA)-ZnPc/Dox	Fruc	ester bond	DOX	3T3, MCF-7, MDA-MB-231, MTT assay, in vitro	Free DOX for 4h 3T3 IC_50_: 22.31 ± 3.39 μg/mL MDA-MB-231 IC_50_: 28.22 ± 3.55 μg/mL GNPs-ZnPc/Dox for 4 h 3T3 IC_50_: 13.21 ± 1.39 μg/mL MDA-MB-231 IC_50_: 10.57 ± 1.27 μg/mL with the presence of light irradiation 3T3 IC_50_: 3.502 ± 0.22 μg/mL MDA-MB-231 IC_50_: 1.43 ± 0.09 μg/mL	pH-sensitive glycopolymer, GLUT5 transporter (fructose transporter)	[236]
14.	P(MAFruc)-*b*-P(3-VBA)-*co*- MMA	Fruc	ester bond imine linker-AMF 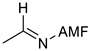	AMF	MCF-7, and MDA-MB-231, SRB assay, in vitro	free amonafide: MCF-7 IC_50_: 11.23 μM MDA-MB-231 IC_50_: 13.98 μM Glycopolymer: MCF-7 IC_50_: 7.19 μM MDA-MB-231 IC_50_: 4.92 μM	GLUT transporters	[247]
15.	P(1-*O*-MA’Fruc)-*b*-PMMA	Fruc	ester bond	DOX	MCF-7, MDA-MB-231, flow cytometry, in vitro	-	GLUT transporters	[253]
16.	P(BOB-HA)-P(Fruc)-PDS/Vc	Fruc	ester bond	DOX	4T1, MTT assay, in vitro	-	light-responsive glycopolymer (NIR)	[242]
17.	PEG-*b*-PAEG-*b*-PAA cl-micelles/Cys	Gal	ester bond	DOX	HepG2, NIH3T3 MTT assay, in vitro	cell viability (%) for 24 h HepG2: 38% NIH3T2: 68%	ASGP-R receptors, redox-sensitive micelles	[254]
18.	PMAGal- * b * -PMAChols	Gal	ester bond	DOX	SK-Hep-1, MTT assay, in vitro	SK-Hep-1 IC_50_: 9.06 μM	receptor ASGP-R	[255]
19.	p(IVDG-*co*-BMDO)	Gal	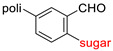	DOX	L929, HeLa, MTT assay, in vitro	free DOX: HeLa IC_50_: 0.8 mg/mL glycopolymer: HeLa IC_50_: 1.9 mg/mL,	pH-sensitive polymeric micelles	[237]
20.	IGPC	Gal	ester bond	DOX	HepG2, MTT assay, in vitro	free DOX: HepG2 IC_50_: 0.45μg/mL DOX-loaded glucose HepG2 IC_50_: 0.75 μg/mL galactose-containing micelles HepG2 IC_50_: 0.20μg/mL	uptake by ASGP-R	[256]
21.	mPEG-*b*-PMAGal-*co*-DOX	Gal	ester bond	DOX	HepG2, MCF-7, MTT assay, in vitro	Free DOX: HepG2 IC_50_: 0.61 μM MCF-7 IC_50_: 0.70 μM Glycopolymer: HepG2 IC_50_: 1.22 μM MCF-7 IC_50_: 2.97 μM	receptor ASGP-R	[257]
22.	(PCL)_2_−*b*-Pr-gly−*b*-GP	Gal	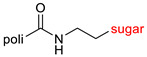	DOX	HepG2, MTT assay, in vitro	free Dox: HepG2 IC_50_: 2.2 μg/mL Dox-loaded UCL (uncross-linked) micelles: HepG2 IC_50_: 5.7 μg/mL Dox-loaded ICL (interface crosslinked) micelles: HepG2 IC_50_: 7.83 μg/mL	receptor ASGP-R	[258]
23.	PMAG-*b*-PAA	Gal	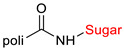	PTX	A549, MCF-7 in vitro	PTX-LANS^®^ (commercially available formulation with PTX) A549 IC_50_: 2 ng/mL MCF-7 IC_50_: 4 ng/mL PMAG-*b*-PAA NPs A549 IC_50_: 1.8 ng/mL MCF-7 IC_50_: 8 ng/mL	-	[259]
24.	PADGal	Gal	ester bond	DOX	HepG2 and HeLa, NIH3T3, MTT assay, in vitro	HepG2 IC_50_: 2.9 μg/mL HeLa IC_50_: 9.0 μg/mL NIH3T3 IC_50_: 12.5 μg/ml	uptake by ASGP-R	[260]
25.	P(MAGal-*co*-DMAEMA)-*b*-PPDSMA	Gal	ester bond	DOX	HepG2, MTT assay, in vitro	-	receptor ASGP-R	[261]
26.	pGal(Ac)-*b*-pNIPAA	Gal	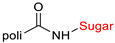	DOX	HeLa, A549, HepG2, MTT assay, in vitro	-	uptake by ASGP-R	[262]
27.	P(AzoMA)-*b*-P(GalEtMA))	Gal	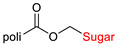	hydrophobic compound Nile red	A375, SRB assay, in vitro	-	light-responsive glycopolymer	[240]
28.	P((DEGMA)-*b*-P(OVNG))	Gal	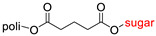	AuNRs	L-929, MTT assay HepG2, flow cytometry, in vitro	-	thermoresponsive glycopolymer (photothermal treatment for the tumor- phototherapy)	[245,246]
29.	PHML-*b*-PMAGal P(HML-*st*-MAGal)	Gal	ester bond	pDNA	H1299, MTT assay, in vitro	-	pDNA binding affinities	[263]
30.	PCL- * b * -PManEA	Man	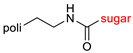	DOX and ConA	UMUC3, MTT assay, in vitro	free DOX UMUC3 IC_50_: 0.79 µg/mL Glycopolymer UMUC3 IC_50_: 1.98 µg/mL	ConA receptor	[264]
31.	P(ManMac)-*r*-(MAA)	Man	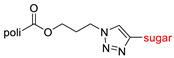	AuNPs with DOX	HeLa, A549, SH-SY5Y, MTT assay, in vitro	-	pH-sensitive drug	[238]
32.	Man-GP-(PCL)2	Man	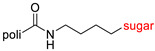	Nile red or Rhodamine B	MDA-MB-231, MTT assay, in vitro	-	receptor MRC2	[265]
33.	PAAMAM- (FUDR+CARB)-MOF-808	Man	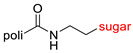	FUDR and CARB	MCF-7, PANC-1, HepG2, cytotoxicity assay, in vitro	free CARB MCF-7 IC_50_: 59.4 μg/mL HepG2 IC_50_: 15.8 μg/mL free FUDR PANC-1 IC_50_: 20.0 μg/mL HepG2 IC_50_: 14.0 μg/mL PAAMAM- (FUDR+CARB)-MOF-808 HepG2 IC_50_: 0.13 (equiv. FUDR μg/mL); 2.4 μg/mL (equiv. CARB μg/mL) MCF-7 IC_50_: 0.46 μg/mL (equiv. FUDR μg/Ml); 8.3 equiv. CARB μg/mL) PANC-1 IC_50_: 0.38 μg/mL (equiv. FUDR μg/Ml); 6.8 μg/mL (equiv. CARB μg/mL)	mannose receptors CD206	[266]
34.	GP’-*b*-PCL	Lac	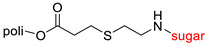	DOX	HepG2, MTT assay, in vitro	Glycopolymer HepG2 IC_50_:0.43 µg/mL DOX-loaded non-glycomicelles HepG2 IC_50_: 6.55 µg/mL	uptake by ASGP-R	[239]
35.	pDMSN-pLAMA	Lac	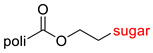	MSNs	HepG2, NIH3T3, MTT assay, in vitro	pDMSN-pLAMA HepG2 IC_50_:0.43 µM	receptor ASGP-R	[267]
36.	P(AcGlcMA-*b*-MAA) P(AcFrucMA-*b*-MAA)	Glu and Fruc	ester bond	DACP-Pt drug	A2780, MCF-7 and MB-MDA-231, flow cytometry, in vitro	DACP-Pt drug: A2780 IC_50_:5.8 µM MCF-7 IC_50_:5.08 µM MB-MDA-231 IC_50_:11 µM Glu polimer: A2780 IC_50_: 2.8 µM MCF-7 IC_50_: 20.1 µM MB-MDA-231 IC_50_: 15.9 µM Fruc polymer:A2780 IC_50_: 2.5 µM MCF-7 IC_50_:7.3 µM MB-MDA-231 IC_50_:4.8 µM	GLUT transporters	[268]
37.	DEGMA-*co*-OVNGmix	Glu Gal	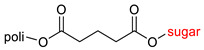	AuNPs and ConA	HepG2, L929, CCK8 assay, in vitro	-	ConA receptor	[269]
38.	HA-(PEG-DNP)	glucuronic acid *N*-acetylglucosamine	-	-	MDA-MB-23, ADCC assay, in vitro	-	multivalent antibody recruiting glycopolymers (MARGs)	[270]
39.	PLAMA-*b*-PSBMA-*b*-PNIPAM	Lac and Gal	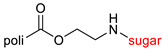	DOX	HepG2,HeLa, CCK-8, in vitro	Nanomedicines with galactose HepG2 IC_50_: 0.31 µg/mL HeLa IC_50_: 1.21µg/mL Nanomedicines without galactose HepG2 IC_50_: 1.42 µg/mL HeLa IC_50_: 1.57 µg/mL	receptor ASGP-R, thermo- and redox-sensitive glycopolymer	[243]
40.	BGP	Fuc, SialA, HD	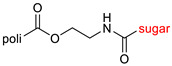	no drug ^a^	COS7, B16, MTT assay, in vitro	-	mimic natural glycosaminoglycan (heparin)	[271]

^a^ no drug -heparin saccharide to mimic the structural characters and biological activities of heparin. Glu: glucose, Gal: galactose, Fruc: fructose, Man: mannose, Lac: lactose, Fuc: fucose, SialA: sialic acid, HD: heparin saccharides. AcGalEtMA: 2-(2,3,4,6-tetra-*O*-acetyl-β-D-galactopyranosyl)ethylmethacrylate, AEG: 2-acryloxyethyl-galactose, AG: 2-(acrylamido)glucopyranose, AzoMA: azobenzene methacrylate, BEMA: 2-(4-formylbenzoyloxy)ethylmethacrylate, BGP: brush glycopolymer, BMDO: 5,6-benzo-2-methylene-1,3-dioxepane, BOB-HA: benzoxaborole modified hyaluronic acid, DEA: 2-(diethylamino)ethyl methacrylate, DEGMA: diethylene glycol methacrylate, DMAEMA: *N*,*N*-dimethylaminoethyl, DNP: dinitrophenol, FMA: furfuryl methacrylate, FruMA: fructomethacrylate, GAMA: gluconamido ethyl methacrylate, GP: glycopolymer, GP’: hydrophilic glycopolypeptide, HA: hyaluronic acid. HEMA: hydroxyethyl methacrylate), IGPC: 1,2;3,4-di-*O*-isopropylidene-3-*O*-MCDO-D-galactopyranose, IVDG: 1,2:3,4-di-*O*-isopropylidene-6-*O*-(2′-formyl-4′-vinylphenyl)-D-galactopyranose, LAMA: 2-lactobionamidoethyl methacrylamide, MAA: methacrylic acid, MAChols: 6-cholesteryloxyhexyl methacrylate, MAEBA: (2-methacryloxyethoxy) benzaldehyde, MAFruc: methacryloyl-D-fructopyranose, MAG: 2-deoxy-2-methacrylamido-D-glucose, MAG’: 3-*O*-methacryloyl-D-glucopyranose, MAGal: 6-*O*-methacryloyl-D-galactopyranose, MCDO: 5-methyl-5-carboxyl-1,3-dioxan-2-one, M-DMSN: magnetic mesoporous silica nanoparticles, MMA: methyl methacrylate, mPEG: methpoy-poly(ethylene glycol), NIPAM: *N*-isopropylacrylamide, OVAG: 6-*O*-vinyladipoyl-D-glucose, OVNGmix: poly(6-*O*-vinyladipoyl-D-galactose or -D-glucose), PAA: poly(acrylic acid), PAAMAM: acrylic acid-mannose acrylamide, PADGal: poly(6-*O*-acryl-D-galactose), PBAE: β-aminoester, PCL: poly(ε-caprolactone), PDS: photosensitiser perylene diimide zwitterionic polymer, PDSMA: pyridyl disulfide ethyl methylacrylate, PEG: poly(ethylene glycol), pGal(Ac): *N*-(prop-2-enoyl)-β-D-peracetylated galactosamine, pNIPAA: *N*-isopropyl acrylamide, PS: poly(styrene), SBMA: sulfobetaine methacrylate, VBA: vinylbenzaldehyde, Vc: ascorbic acid.

#### 3.2.3. Sugars Incorporated into Glycopolymers

##### Glucose Glycopolymers

Glucose polymers are used to deliver drugs, such as DOX [232,234,235,241], PTX [231], BTZ [233], and CDDP [248]. Glucose cannot cross cell membranes by simple diffusion, but glucose has been noted to accumulate in many human tumor types. It is reasonable to conclude that GLUT-1 are present in tumor vasculature to meet the high glucose demand of tumor cells, an important target for the strategy to improve targeted transport [272]. It is suspected that glucose-containing polymers have a mechanism of targeting GLUT-1 transporters and, therefore, can be targeted to tumors where GLUT-1 receptors are overexpressed. This is, for example, the cisplatin-containing polymer, shown in Table 4, Entry 8, targeting human squamous carcinoma cells OSC-19. GLUT-1-targeted carriers were investigated as a new strategy targeted at overcoming the vascular barrier, increasing delivery and efficacy in tumors. These nanodrugs were prepared by controlled addition of glucose to polymeric micelles containing the anticancer agent cisplatin. Micelles loaded with cisplatin that served as carriers for this study showed strong anticancer effects and reduced side effects in humans [248].

##### Fructose Glycopolymers

Fructose glycopolymers are taken up by cell lines that overexpress the GLUT-5 transporter. The GLUT-5 transporter is not a lectin, but a transport protein that allows the entry of fructose into cells. GLUT-5 has been found to be overexpressed in breast cancer. The presence of fructose in polymer carriers enhances uptake by breast cancer cells, such as MCF-7 and MDA-MB 231. They have been used in glycopolymer carriers with anticancer activity with drugs such as DOX [236,242,252,253], PTX [250], CDDP and GEM [252], curcumin [249], and AMF [247].

##### Galactose Glycopolymers

There are many examples in the literature of targeting asialoglycoprotein receptors (ASGP-R) with glycopolymers. ASGP-R is a C-type lectin, selective for galactose, found in many liver cells such as Hep-G2 [245,246,256,257,258,260]. The role of ASGP-R is to remove asialoglycoproteins by recognizing galactose or galactosamine present on their surface, and this is a calcium ion-dependent process. Due to this mechanism, galactose-containing glycopolymers can be delivered to the tumor cells drugs, such as DOX [237,254,255,256,257,258,260,262], PTX [259], pDNA [259], and AuNRs [245,246].

An interesting example of a galactose glycopolymer is P(HML-MAGal) (Table 4, Entry 29). The cationic block copolymer PHML-b-PMAGal and the statistical copolymer P(HML-st-MAGal) with side moieties of natural galactose and (L)-lysine showed high binding affinity for plasmid DNA. The MTT test showed that the glycopolymers had lymph node-derived cytotoxicity against the non-small cell lung cancer cell line (H1299); the amount of cytotoxicity was dependent on the architecture of the block/random polymer and the galactose content. Furthermore, it could be seen that the random copolymer P(HML_40_-st-MAGal_4_) with ~5% galactose content showed the highest gene transfection efficiency among synthesized cationic glycopolymers. The analysis of the endocytosis pathway showed that the polyplexes P(HML40-st-MAGal4)/pDNAs entered H1299 cells mainly through the endocytosis pathway and pDNAs showed relatively rapid cellular uptake capacity and obvious endosome/lysosome escape effect. These results indicate that these cationic glycopolymers can serve as potential vehicles for safe and efficient gene delivery [263].

Furthermore, galactose polymers can potentially be used to target galectin-3, which is overexpressed in some cell lines, such as the melanoma cell line A375, as was the case light responsive galactose-based glycopolymer (Table 4, Entry 26), which showed selective cytotoxicity in human melanoma cells [240].

##### Mannose Glycopolymers

Mannose receptors, such as MRC2, have been overexpressed only in some tumor cell lines, such as MDA-MB-231 or MCF-7, facilitating cellular uptake of mannose-based polymers. This C-type mannose receptor is overexpressed also on dendritic cells and macrophages [265]. Viruses often use mannose-containing structures to target mannose receptors, type C lectins, so carriers with mannose are most often used for immunotherapy. The mannose-binding lectin receptors CD206 are overexpressed in many cells, including immune cells and liver cancer cells [266].

##### Disaccharide Glycopolymers

Glycopolymer-modified mesoporous magnetic silica nanoparticles, such as M-DMSN@pLAMA constructed by 2-lactobionamidoethyl methacrylate (LAMA) and magnetic mesoporous silica nanoparticles (M-DMSN), are worth mentioning. This carrier for anticancer drug delivery showed high drug loading capacity and improved MR imaging. The nanoparticles, due to the introduction of polymer containing a galactose, were characterized by a high drug loading capacity and were taken up by HepG2 cells [260].

##### Other Glycopolymers

An interesting example of the use of glycopolymers appears to be the hyaluronic acid-based, dinitrophenol-grafted glycopolymer described by the acronym HA-[PEG3-DNP] (Table 4, Entry 38), which has shown the ability to recognize cells containing the CD-44 antigen. This polyvalent glycopolymer showed an antibody recruiting ability and anticancer activity in in vitro studies. Thus, it can be speculated that it may be successful in cancer therapy when used as an immunotherapeutic agent [270].

Drug delivery studies of glycopolymers obtained using one type of sugar target one specific receptor. However, a combination of the two saccharides to target the two surface receptors may be beneficial. Nanocarriers prepared from a blend of block copolymers containing the two sugars can be supplied by two mechanisms, and an enhanced targeting effect is possible. For example, thermosensitive and redox-sensitive star-shaped nanogels are beneficial in this process (Table 4, Entry 39). The thermosensitive and redox-sensitive core consists of disulfide bonds cross-linked poly(N-isopropylacrylamide) (PNIPAM), poly(sulfobetaine methacrylate) (PSBMA) and the lactose moiety, poly(2-lactobionamidoethyl methacrylamide) (PLAMA). (PLAMA-b-PSBMA)-b-PNIPAM nanogels reinforced DOX delivery in HepG2 cells as a result of the specific binding of the lactose residue to ASGP-R. The IC_50_ value of DOX-loaded nanogels was significantly lower in human hepatoma cells (HepG2) compared to non-hepatic HeLa cells. DOX uptake by HepG2 cells varied depending on the presence of galactose. When galactose was added to the nanocarrier, the IC_50_ value was even lower. This indicates a positive effect of using two saccharides in this type of carrier and shows promising results for the selective delivery of drugs to hepatoma cells [243]. The new brush glycopolymer (BGP), in combination with three different sugars—sialic acid, fucose, and heparin disaccharides (Table 4, Entry 40), has the ability to mimic natural glycosaminoglycans such as heparin, the PSGL-1 P-selectin ligand, resulting in it having great potential to inhibit cancer metastasis. In a mouse metastatic melanoma model, this glycopolymer inhibited B16 cell metastasis [271].

## 4. Calculation Methods in Drug Design

Drug design is a considerable challenge for researchers because it is a complicated and time-consuming process, and the final effect depends on a number of important factors. In the case of searching for new bioactive substances and designing their semi-synthetic analogs with therapeutic potential, the synthesis and then experimental determination of pharmacokinetic and pharmacodynamic parameters is a long and expensive process.

The effectiveness of therapeutic agents is closely related to their bioavailability, which in turn correlates with the chemical structure. An ideal drug candidate should have a good safety profile and a balance between appropriate potency, pharmacokinetic properties, and ADME parameters (Absorption, Distribution, Metabolism, Excretion).

Unsatisfactory pharmacokinetics and high toxicity detected in the late stages of drug design (only during in vitro and in vivo studies) are one of the main obstacles to further development of the substance as a drug candidate. Therefore, an important part of the entire research process should be computational methods (e.g., QSAR), which enable preliminary research for a drug designed from scratch or verification and simulation of potential properties in the case of designing molecular hybrids of a known bioactive substance.

There are several rules for determining the similarity of chemicals with drugs. For example, compounds with good oral absorption are defined by the ‘Rule of Five’ (RoF) formulated by Lipinski [273,274]. This is the first and still widely used rule, the basic guidelines of which are the values of the following parameters: logarithm of the octanol: water coefficient (lipophilicity, log P < 5; MW < 500 Da; number of H-bond donors (HBDs) < 5; number of H-bond acceptors (HBAs) < 10). Another criterion to predict the drug-likeness of molecules is the estimation of their molecular descriptors, e.g., molecular weight (M), topological polar surface of the molecule (tPSA), nRB (number of rotatable bonds), and brain/blood partition coefficient (log BB) [275]. The use of computational techniques and molecular modeling significantly reduces costs, shortens the time spent on research on a new drug, and above all, minimizes the risk of failure.

After the discovery of the anticancer activity of cisplatin, one of the most effective drugs currently used, computational studies were carried out in parallel as a supplement to experimental work. Computational models have greatly contributed to understanding of the hydrolysis process that activates cisplatin, as well as the preferential anticancer activity of the *cis* isomer over the *trans* isomer. They also enabled the determination of cisplatin–DNA interactions and the design of new analogs [276].

Additionally, in the case of paclitaxel, the development of cancer cell resistance and serious side effects in patients require further improvement of the drug. Structure–activity relationship studies (SAR) in search of PTX analogs with higher activity against cancer cells and lower toxicity against normal tissues have been conducted since the 1980s and have been widely described in many papers [68,277,278,279].

In 2023, Amin’s group designed and synthesized thirteen 8-hydroxyquinoline conjugates. Activity research anticancer treatment was preceded by in silico predictions of physicochemical parameters and estimation of pharmacokinetic properties [280].

The in silico approach can also be used to predict the synergism of different drug combinations (eg, PTX, DOX, and 5-FU) and evaluate the pharmacokinetic parameters of combinations of peptides and anticancer agent to analyze interactions in vitro. Peptide combinations with PTX and DOX have been shown not to be effective against MCF-7 and PC-3 cancer cells compared to drug treatment. However, as expected in silico, the combination of peptides with 5-FU produced synergistic cytotoxic effects against HT-29 colorectal cancer cells [281].

Unfortunately, there are many steps and obstacles that a compound must overcome in order to become an effective drug. It should be remembered that indicating the correct pharmacokinetic parameters and ensuring bioavailability and safety profile does not guarantee success. Moreover, the calculation methods will not replace in vitro and in vivo tests, but they can significantly support scientists at the stage of designing structural changes intended to improve the drug properties.

## 5. Conclusions

In this review, we draw attention to the problems associated with the imperfections of commonly known and used anticancer drugs, which cause the occurrence of numerous side effects of therapy with their use. Since cancer is a huge problem in the 21st century, much attention is paid to this topic. There is a huge amount of scientific papers describing proposals to improve the properties of existing drugs and obtain new ones based on compounds showing anticancer activity. One of the possibilities to improve the effectiveness and safety of chemotherapeutic agents is the development of targeted therapies that will allow the drug to selectively interact with cancer cells. The so-called targeted drugs can be obtained by temporarily ‘turning off’ the activity of the drug by obtaining a physiologically stable prodrug that is able to reach the target site and only there disintegrates, releasing the active substance. This is possible by attaching an appropriate ligand to the drug, which will be recognized by cancer cells. Another possibility is the use of various types of carriers that enable controlled release of the drug under the influence of factors characteristic of the tumor microenvironment. Such a carrier is often labeled with a suitable ligand that allows it to be targeted to cancer cells. In both discussed cases, such a ligand should target unique features of the tumor, e.g., lectins and protein transporters, that are overexpressed in certain types of cancer.

**Figure 15:**
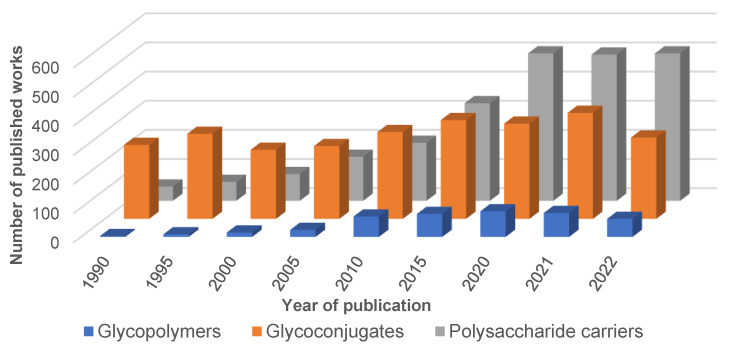
Publications per year on selected sugar applications, based on a *Scopus* search using the keywords ‘glycopolymers’, ‘glycoconjugates’, and ‘polysaccharide carriers’ (access date: 15 January 2023).

There are many reports in the literature on the use of sugars to target drugs to cancer cells. In general, interest in glycoconjugates, glycopolymers, and polysaccharide drug carriers has remained at a relatively high level for years (as in the case of glycoconjugates) or is even growing all the time, which is especially visible in the case of polysaccharide drug carriers (Figure 15).

Due to the extensiveness of the topic discussed, we focused only on selected examples that, in our opinion, will allow the reader to see the huge potential that lies in the use of broadly understood sugars to obtain effective and selective anticancer pharmaceuticals. The discussed examples show the benefits of the selected modifications so clearly that maybe they will become a stimulus for further intensive exploration of the topic related to the use of sugars to improve the properties of compounds used to fight one of the most important civilization diseases of the 21st century.

## Data Availability

Not applicable for this type of publication. All data are available in references.

## Abbreviations

**Anticancer drugs**	
5-FU	5-fluorouracil
ADM	adriamycin
AMF	amonafide
BTZ	bortezomib
CARB	carboplatin
CDDP	cisplatin
CLB	chlorambucil
ConA	concanavalin A
DHA	docosahexaenoic acid
DOX	doxorubicin
DTX	docetaxel
GEM	gemcitabine
MTX	methotrexate
PDX	podophyllotoxin
PTX	paclitaxel
**Cell lines**	
4T1	murine breast cancer cell line
A375	human melanoma cell line
A549	human pulmonary adenocarcinoma cell line
A2780	epithelial ovarian cancer cell line
A2780/CP	CDDP-resistant subline
AsPC-1	human pancreas adenocarcinoma
B16-F1	mouse melanoma cell line
B16-F10	mouse melanoma cell line
BJ-H-tert RPMI	normal fibroblasts
C26	murine colorectal carcinoma cell line
Calu-3	human lung cancer cell line
C57BL6	mice melanoma cell line
CEM	T-lymphoblastic leukemia cell line
Colo-205	colorectal adenocarcinoma
CT26	mouse colon adenocarcinoma cell line
DLD-1	colorectal adenocarcinoma cell line
G 361	malignant melanoma cell line
H1299	human non-small lung carcinoma cell line
HaCaT	keratinocyte cancer cell line
HCT-116	human colon carcinoma cell line
HeLa	human cervical cancer cell line
HepG2	human hepatocellular carcinoma cell line
HL-60	human promyelocytic leukemia cell line
HTB-177	lung cancer cell line
HUVEC	human umbilical vein endothelial
Hs683	human brain glioma cell line
K-562	human chronic myelogenous leukaemia
MCF-7	human breast cancer cell line
MDA-MB-231	human breast cancer cell line
NCI-H460	lung cancer cell line
CD44^-^	fibroblast cell line
NKE	normal kidney epithelial cell line
OVCAR-3	ovarian carcinoma cell line
OVCAR-8	ovarian carcinoma cell line
PANC-1	human pancreas ductal adenocarcinoma
PC-3	human prostate cancer cell line
RPMI 8226	multiple myeloma cell line
22Rv1	human prostate carcinoma cell line
Saos-2	human osteosarcoma cell line
SHY5Y	hyman neuroblastoma cell line
SK-Hep-1	human hepatic adenocarcinoma
SGC-790	endocervical adenocarcinoma
U-251	human glioblastoma cell line
WS1	normal skin fibroblasts
VMCF-7	human breast cancer
XHepG2	human liver cancer cell line
